# High level of circulating cell-free tumor DNA at diagnosis correlates with disease spreading and defines multiple myeloma patients with poor prognosis

**DOI:** 10.1038/s41408-024-01185-6

**Published:** 2024-11-28

**Authors:** Marina Martello, Vincenza Solli, Gaia Mazzocchetti, Antonio Giovanni Solimando, Davide Bezzi, Barbara Taurisano, Ajsi Kanapari, Andrea Poletti, Enrica Borsi, Silvia Armuzzi, Ilaria Vigliotta, Ignazia Pistis, Vanessa Desantis, Giulia Marzocchi, Ilaria Rizzello, Lucia Pantani, Katia Mancuso, Paola Tacchetti, Nicoletta Testoni, Cristina Nanni, Elena Zamagni, Michele Cavo, Carolina Terragna

**Affiliations:** 1 Department of Medical and Surgical Sciences (DIMEC), University of Bologna, Bologna, Italy; 2 IRCCS Azienda Ospedaliero-Universitaria di Bologna, Istituto di Ematologia “Seràgnoli”, Bologna, Italy; 3 Department of Precision and Regenerative Medicine and Ionian Area (DiMePRe‑J), Unit of Internal Medicine “Guido Baccelli”, University of Bari “Aldo Moro” Medical School, Bari, Italy; 4 Nuclear Medicine Unit, AUSL Romagna, Ravenna, Italy; 5 Department of Precision and Regenerative Medicine and Ionian Area (DiMePRe‑J), Section of Pharmacology, University of Bari “Aldo Moro” Medical School, Bari, Italy; 6 Nuclear Medicine, IRCCS Azienda Ospedaliero-Universitaria di Bologna, Bologna, Italy

**Keywords:** Disease-free survival, Cancer genomics, Myeloma

## Abstract

Multiple myeloma (MM) is a plasma cell (PC) disorder characterized by skeletal involvement at the time of diagnosis. Recently, cell-free DNA (cfDNA) has been proven to recapitulate the heterogeneity of bone marrow (BM) disease. Our aim was to evaluate the prognostic role of cfDNA at diagnosis according to disease distribution, and to investigate the role of the MM microenvironment inflammatory state in supplying the release of cfDNA. A total of 162 newly diagnosed MM patients were screened using 18F-FDG PET/CT and assessed by ultra low-pass whole genome sequencing (ULP-WGS). High cfDNA tumor fraction (ctDNA) levels were correlated with different tumor mass markers, and patients with high ctDNA levels at diagnosis were more likely to present with metabolically active paraskeletal (PS) and extramedullary (EM) lesions. Moreover, we demonstrated that microenvironment cancer-associated fibroblast (CAFs)-mediated inflammation might correlate with high ctDNA levels. Indeed, a high cfDNA TF level at diagnosis predicted a poorer prognosis, independent of R-ISS III and 1q amplification; the inclusion of >12% ctDNA in the current R-ISS risk score enables a better identification of high-risk patients. ctDNA can be a reliable and less invasive marker for disease characterization, and can refine patient risk.

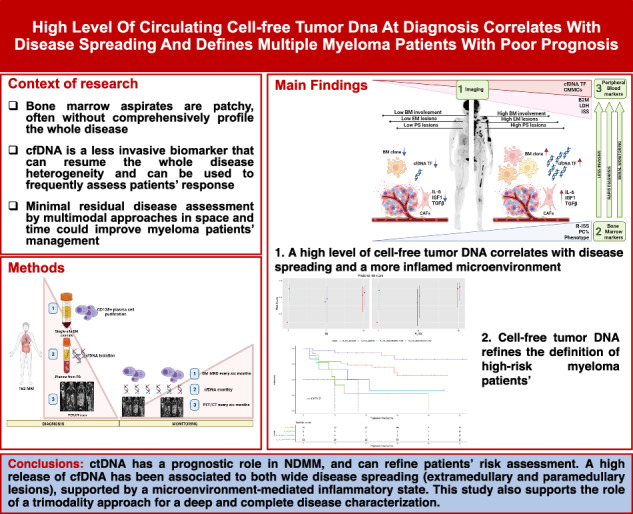

## Introduction

The recent history of multiple myeloma (MM) has been marked by remarkable advances in available treatments, which have ultimately improved patient survival [1, 2]. Boosting and redirecting the immune system against the tumor clone using monoclonal antibodies, antibody-drug conjugates, CAR-T cells, and bispecific antibodies led to deeper and long-term disease remissions [3, 4]. For this reason, monitoring of disease dynamics over time through the measurement of residual bone marrow cells is rapidly gaining attention, both in clinical trials and in daily practice, aiming at the prompt prevention of any disease reappearance [5, 6].

However, a single bone marrow biopsy may not be fully representative of the entire disease heterogeneity. Indeed, MM is typically characterized by patchy bone marrow plasma cell infiltration and nodular proliferation of plasma cells, giving rise to the so-called focal lesions (FL) detected by imaging techniques, such as PET/CT [7–9], and the propensity of the disease to spread outside the bone marrow, frequently associated by a spatial heterogeneity [10–12]. Moreover, since MRD negativity needs to be confirmed over time, the invasiveness of bone marrow sampling should not be neglected, as it may not be tolerable when repeatedly performed [13].

For these reasons, in recent years, liquid biopsy has gained an increasingly key role in disease characterization and disease monitoring [14–17]. Circulating cell-free DNA (cfDNA) has already been widely adopted as a surrogate marker for several indications in cancer, including diagnosis, prognosis, and monitoring, by preventing the invasiveness of tissue biopsies [18]; in MM, the role of cfDNA is still under investigation.

cfDNA is the result of cell breakage owing to necrosis, apoptosis, and secretion. Therefore, these small DNA fragments resume the full set of information derived from both normal and tumor cells, thus offering an easily accessible readout of the disease [19–22]. Moreover, the release of cfDNA by the neoplastic clone might be conditioned by the tumor’s surrounding microenvironment [23], suggesting that cfDNA can act either as an immune homeostasis regulator or as a “cancer message deliverer” to other sites.

At present, cfDNA might represent a valuable opportunity both to profile MM disease and possibly improve minimal residual disease assessment by integrating information derived from cfDNA can be read alongside that provided by current standard methods, such as BM biopsy profiling and imaging scans performed by MRI or PET [24, 25].

This study aimed to investigate the biological role of cfDNA in resuming MM disease at diagnosis, both quantitatively and qualitatively, and the possibility of a multilevel minimal residual disease assessment throughout the disease course, integrating cfDNA analysis, BM MRD assessment, and PET imaging. Moreover, we investigated the prognostic significance of cfDNA by assessing its interaction with currently employed risk stratification methods [26, 27]. Finally, we obtained insights into the role of the inflammatory state of the MM microenvironment to provide a possible explanation for the relationship between released cfDNA and the diffuse distribution of the disease.

## Methods

### Participant enrollment and clinical sample collection

**Fig. 1:**
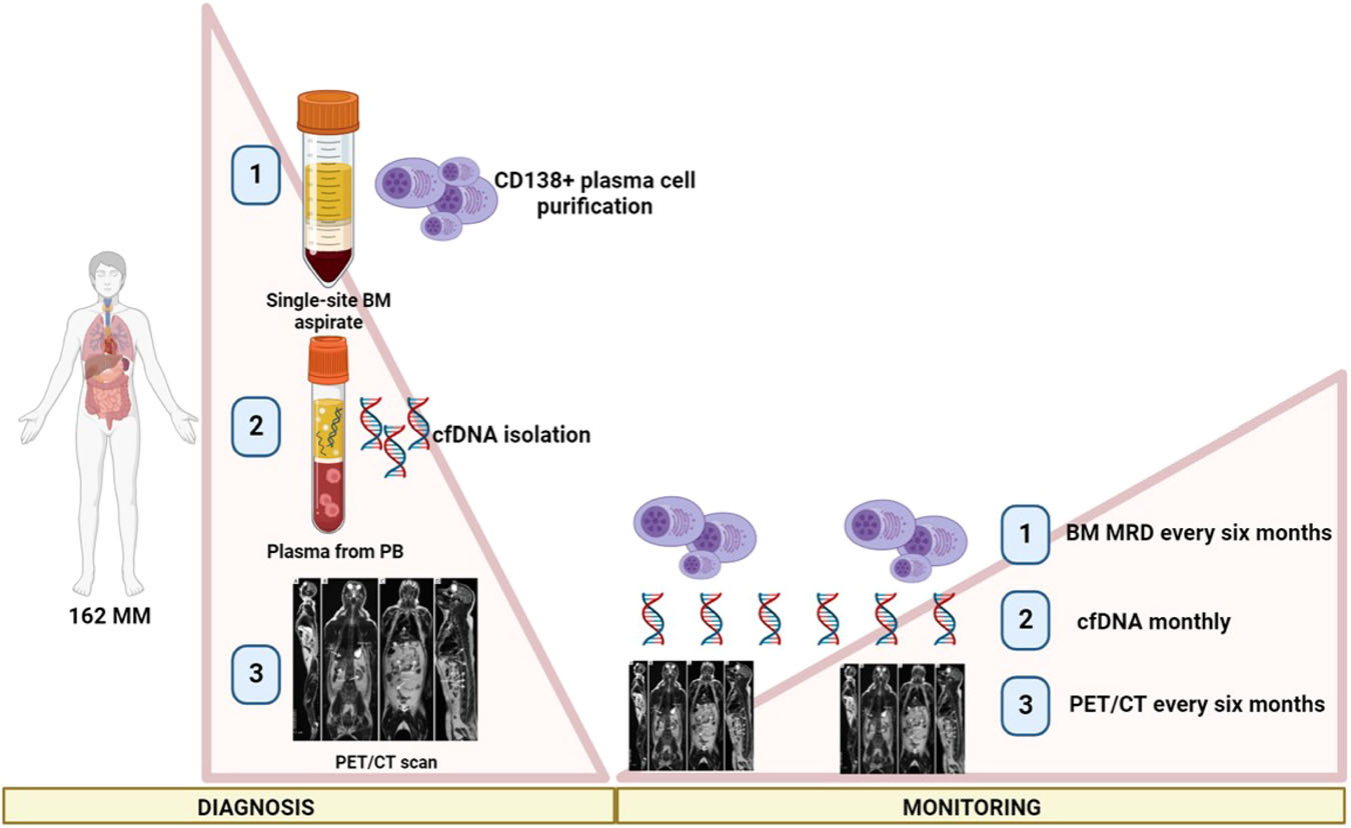
Biological samples’ biobanking and timing of MRD monitoring by a trimodality approach. A specific biological samples’ biobanking has been planned which includes bone marrow (BM) samples and peripheral blood (PB) samples at diagnosis for both CD138+ plasma cells purification and cfDNA isolation, respectively. Moreover, a PET/CT scan has been performed at baseline. To monitor the disease, BM MRD and PET/CT scans have been employed as conventional methods. PB has been collected monthly to isolate plasma for cfDNA. By this trimodality approach, a total of 162 patients have been studied at baseline, and for 22 of them, we have monitored the disease after therapy. Created with BioRender.com.

A total of 162 newly diagnosed multiple myeloma patients were enrolled in this study, for whom peripheral blood and bone marrow samples were available (Fig. 1). Informed consent for treatment and sample procurement by the Declaration of Helsinki was obtained for all cases included in the study. The cohort was representative of an overall MM patient population, as shown in Supplementary Tables 1, 2. All the chromosomal alterations were assessed by FISH analyses. All radiographic imaging was performed by 18F-FDG PET-CT (Supplementary Table 3). Informed consent for treatment and sample procurement by the Declaration of Helsinki was obtained for all cases included in the study (AIRC IG2018 “StreaMMing” project approved by ethical committee EC n. 167/2019/Sper/AOUBO).

### Ultra low-pass whole genome sequencing

Ultra low-pass WGS was performed both on cfDNA from plasma and on gDNA derived from BM to identify the gDNA and cfDNA tumor fractions and the grade of similarity between the two tissues. Whenever ctDNA is mentioned in the text, reference is made to cfDNA tumor fraction.

### Cell purification and cultures

BM mononuclear cells (BMMCs) were isolated from heparinized BM aspirates via Ficoll–Hypaque gradient separation. Bone marrow stromal cells (BMSCs) were obtained after the adhesion of BMMCs to polystyrene flasks and cultured in DMEM with the addition of 1% penicillin/streptomycin and 10% fetal bovine serum. Cancer-associated fibroblasts (CAFs) were isolated from BMSCs through D7-FIB-conjugated (anti-fibroblasts) microbeads and cultured with DMEM supplemented with 1% penicillin/streptomycin (Euroclone) and 20% FBS. Total RNA was isolated from 1 × 10^6^ BMSCs and CAFs using the RNeasy Mini kit and reverse-transcribed into total cDNA with the iScript cDNA Synthesis kit according to the manufacturer’s instructions.

### Immunofluorescence staining, adhesion assay, and human cytokine array

BMSCs (1 × 10^5^) were cultured onto microscope slides suitable for microscopy until they reached the desired confluence. Fixation and staining of IL-6 have been described more in detail in the Supplementary files. MM1S were stained with Calcein-AM for 1 h, and then plated in triplicate in 96-well on CAFs isolated from cfDNA low and cfDNA high patients (ratio= MM1S:CAFs, 2:1). After 24 h, non-adherent cells were washed away and the rate of adherent cells was evaluated reading fluorescence at 495 nm by VICTOR™ X3 Multilabel Plate Reader. BM plasma and cell lysates from fresh purified CAFs were analyzed using the Proteome Profiler Human Cytokine Array Kit according to the manufacturer’s instructions. Densitometric quantification of the resulting membranes was performed using Kodak Molecular Imaging Software, and the average pixel density of each protein was normalized to reference spots.

### Bioinformatic and statistical analyses

FASTQ files were obtained and analyzed using MultiQC [28] to evaluate experimental metrics. BAM files were generated by applying GATK best practices for data preprocessing, including read mapping to the reference genome. BAM files were then processed through IchorCNA [29]. CN values were estimated from the average signal and corrected for the sample ploidy obtained by BoBafit [30]. The cfDNA cutoff predictive for prognosis in terms of PFS and OS has been determined through a receiving operating characteristics (ROC) curve. Comparisons between patient groups were performed using Pearson’s *χ*^2^ test or Fisher’s exact test for categorical data and the Kruskal–Wallis test for continuous data. The %CV (standard deviation/mean*100) was also used to assess variability. Survival analyses have been defined according to Kaplan-Meier survival curves (PFS event is defined as disease progression or death).

## Results

### ctDNA quantitatively mimics the BM disease burden and reflects the genomic complexity of neoplastic clones

As ctDNA can be more strictly related to MM tumor burden than total cfDNA, we further investigated this parameter, by measuring the abundance of CNVs carried by cfDNA, namely tumor fraction.

**Fig. 2:**
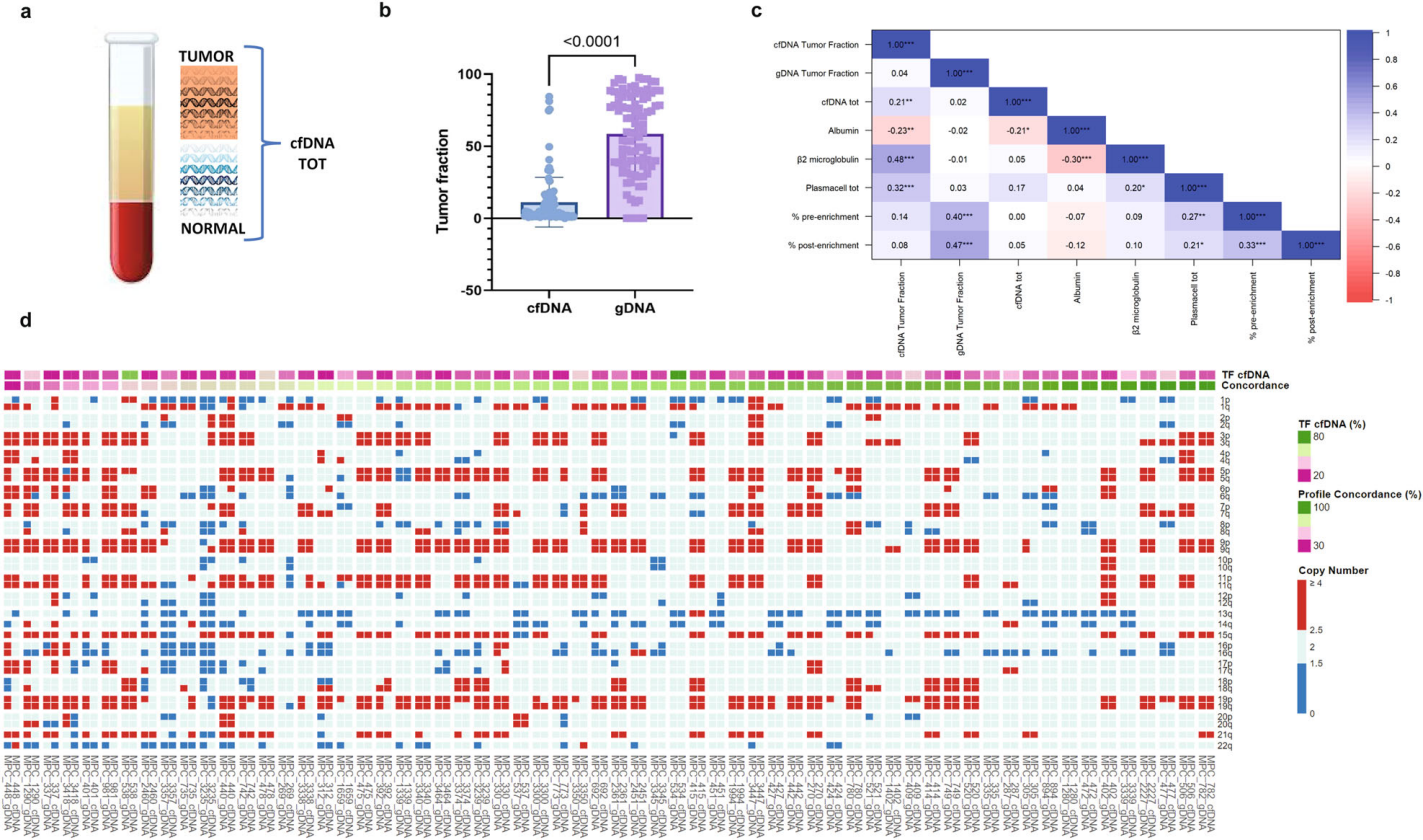
cfDNA tumor fraction as a surrogate of tumor mass index and proxy of genomic complexity. **a** Cartoon representing that cell-free DNA included both DNA fragments from normal cells (total cfDNA) and fragments from tumoral cells (ctDNA). **b** Box plot representing cfDNA tumor fraction (ctDNA TF) median amount in the blood as compared to median gDNA TF in the bone marrow: ctDNA TF is significantly lower than gDNA TF; however, they are strictly correlated. **c** Correlation matrixes demonstrating that ctDNA is correlated to some MM tumor mass markers, with significant correlation observed for b2-microglobulin, albumin, and total bone marrow plasma cells, similarly observed for the gDNA tumor fraction. Conversely, the total cfDNA is less correlated with tumor mass since it can depend on other non-disease-related factors. **d** Brick plot illustrating the comparison between the CNVs profiles derived both from BM gDNA and from cfDNA, by two-site analysis, to investigate the degree of similarity between the two tissues. A percentage of concordance between cfDNAs and gDNAs clonal CNVs was calculated, as expressed by the ratio between the number of concordant segments and the total segments’ number: most patients displayed high concordance between CNVs, as identified in both tissues (46/62 = 75.4%; concordance >75%), whereas a small proportion of patients had slightly similar genomic profiles (16/62 = 26.2%; concordance <75%).

A wide range of ctDNA values was observed among patients (ctDNA median: 3%, range: 0–99.5%). However, as opposed to what we observed for total cfDNA, the comparison between ctDNA and parameters related to MM tumor mass revealed a significant correlation, particularly for b2-microglobulin (*r* = 0.48, *p* value ≤0.001), albumin (*r* = −0.23, *p* value = 0.006) and total BM plasma cells (*r* = 0.32, *p* value ≤0.001). Similarly, the gDNA tumor fraction (describing the bone marrow neoplastic clone) significantly correlated with the same tumor mass-related markers (Fig. 2).

Recently, cfDNA has been reported to recapitulate the landscape of BM neoplastic clones’ molecular alterations (particularly the mutation profile) in some representative patients [22]. Here, a two-site analysis was employed to systematically compare the CNV profiles derived from both BM gDNA and cfDNA, aiming at the identification of the degree of similarity between the two tissues (Fig. 2). A percentage of concordance was calculated, as expressed by the ratio between the number of concordant segments and the total segment number. We observed that most patients displayed high concordance, as identified in both tissues (46/62 = 75.4%; concordance >75%), while a small proportion of patients had slightly similar genomic profiles (16/62 = 26.2%; concordance <75%). However, we observed that the rate of genomic profile concordance was directly related to the tumor fraction percentage (median ctDNA between the abovementioned subgroups = 12.06 vs. 3.99; *r* = 0.60; *p* = 3.63e^−7^; Supplementary Fig. 1) and, consequently, dependent from the limit of detection of the method (<3%). Nevertheless, it has been possible to observe that 3 out of 16 patients with dissimilar genomic profiles had high ctDNA (ranging from 12.07 to 23.98%).

In summary, ctDNA can quantitatively mimic the tumor burden, similar to the tumor fraction of genomic DNA derived from bone marrow, and overall reflects the BM neoplastic clone genomic complexity, with restriction to clonal copy number alterations.

### ctDNA can resume bone marrow neoplastic clone dynamics

**Fig. 3:**
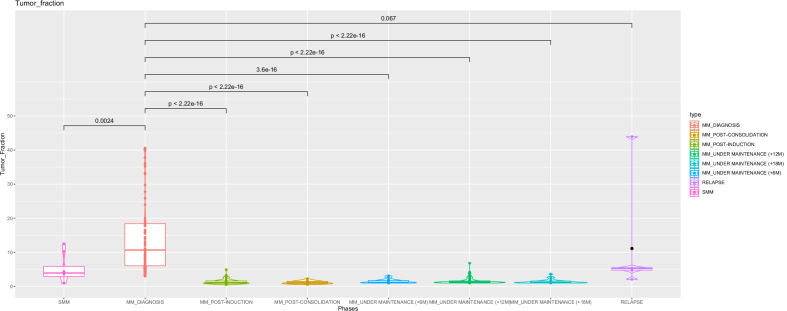
ctDNA can resume the neoplastic clones’ dynamic. cfDNA TF values fluctuation throughout the different disease phases, from SMM to MM and under therapy, post-induction, post-consolidation and under maintenance (three time-points: +6 months, +12 months, +18 months).

As ctDNA can mirror the tumor burden dimension, we aimed to confirm this observation in different clinical phases, namely in preneoplastic phases, where BM tumor burden varies due to its natural evolution, and in MM under treatment (Fig. 3).

SMM patients are commonly characterized by a reduced tumor burden, as compared to that of the neoplastic phase. Indeed, the ctDNA was significantly higher in MM patients than in SMM patients (*n* = 162 vs. 7; median ctDNA: 11.40 vs. 4.22; *p* = 0.0024). Moreover, ctDNA values were much higher in SMM patients who already evolved to MM, than in nonevolved patients (*n* = 4 vs. 3; median cfDNA tumor fraction: 4.22 vs. 1.0; *p* = ns) and, more interestingly, also in patients who had a rapid progression to the neoplastic phases (within 15 months), than those who displayed a slow evolution dynamic (>20 months) (*n* = 2 vs. 2; median ctDNA: 9.70 vs. 2.85; *p* = ns) (Supplementary Fig. 2).

The ability of ctDNA to mimic the BM tumor burden was also confirmed in a subgroup of 22 newly diagnosed MM patients, whose ctDNA dynamics were monitored under therapy, throughout a median of 15 months (range: 1–35), by 271 monthly analyzed time-points. Overall, we observed a tumor fraction decrease during induction therapy (from 11 to 2% median tumor fraction; *p* < 0.05; BM MRD negative at 6th month-time point with sensitivity below 10^−5^ in 21/22 pts), even though some patients displayed peaks of ctDNA release, randomly occurring along the disease course. In general, after a plateau phase, reached approximately under consolidation therapy (median ctDNA = 1.1%; BM MRD negative at 12th month-time point with sensitivity below 10^−5^ in 22/22 pts), the ctDNA remained undetectable in most patients, whereas in a small cohort of patients (3/22, all having cfDNA TF >12% at diagnosis), it started to increase during maintenance, up to 7% TF, within the 18th month of observation (median ctDNA = 1.3%; BM MRD negative at 18th month-time point with sensitivity below 10^−5^ in 20/22; Supplementary Fig. 3; exemplary patients monitored in BM and PB are illustrated in Supplementary Fig. 4 and Supplementary Tables 4, 5).

Therefore, even though more data on an enlarged cohort of patients are needed, here we provide evidence that ctDNA can be reliably employed as a quantitative surrogate marker of BM tumor burden dynamics.

### Identification of a ctDNA cut-off able to improve patient risk stratification

Aiming at the employment of ctDNA as an index of tumor mass, possibly correlated with patient prognosis, we approached a statistical method to define a ctDNA cut-off, that is able to discriminate patients with either high or low tumor fraction.

To this aim, a ROC cut point analysis was employed on 150 pts, for whom clinical data were available, to identify the ctDNA value (ranging from 0 to 99.5%), which best predicted the progression-free survival. A cut-off of 12% tumor fraction was determined (*p* value = 0.0000782; AUC = 0.585; Accuracy = *0*.66; Sensitivity = 0.56; Specificity = 0.73), able to stratify patients into two distinct categories: the first one including 38 patients, characterized by high ctDNA values (range ctDNA = 12–95.5%), and the second one including 112 patients, characterized by low tumor fraction levels (range ctDNA = 0–12%) (Supplementary Fig. 5).

**Fig. 4:**
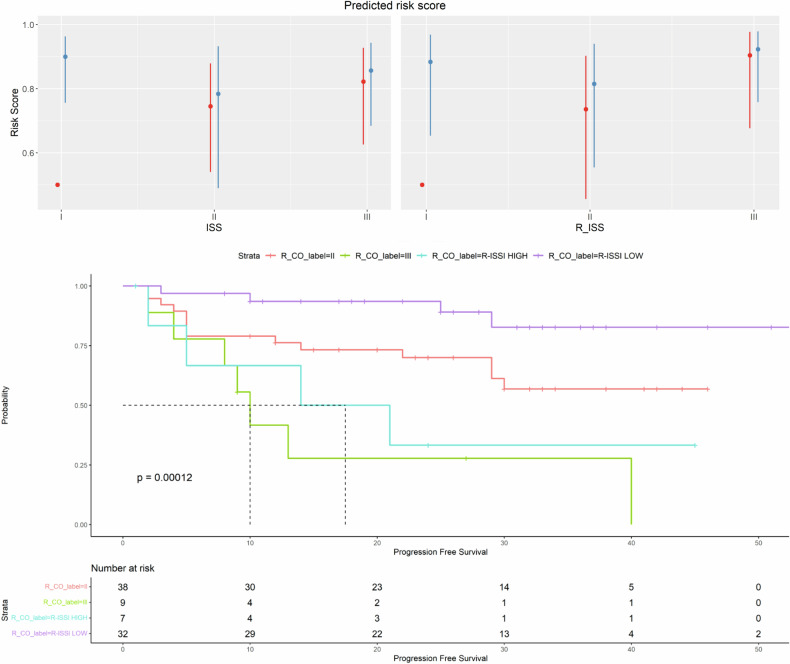
Interaction model between risk scores and ctDNA to determine its contribution to patients' risk status definition. An improved risk definition was achieved by integrating the high-low cfDNA tumor fraction stratification to the R-ISS score levels, highlighting that R-ISS I patients with high ctDNA (i.e., ≥12%) had a progression-free survival time similar to that of R-ISS III patients (R-ISS I high ctDNA vs. R-ISS III median PFS months: 17.5 vs. 10 months; *p* = *ns*). R_CO: variable cut-off >12% ctDNA tumor fraction.

We observed that patients with high ctDNA more frequently carried 1q amplification (*p* = 0.043) and had a low incidence of t(11;14) (*p* = 0.028). To further assess the possible ctDNA contribution to refining the patients’ risk of progression, an interaction model between commonly employed risk scores [23, 24] and ctDNA was explored. Patients with the high release of ctDNA were consistently either ISS III or R-ISS III, and progressively increasing median ctDNA values were measured in patients with ISS and R-ISS risk scores increasing from I to III (*p* value = 0.0019) (Supplementary Fig. 6). Therefore, an improved risk definition was achieved by integrating the high-low ctDNA values stratification to the R-ISS score levels, highlighting that R-ISS I patients with high ctDNA (i.e., ≥12%) had a progression-free survival time similar to that of R-ISS III patients (R-ISS I high ctDNA vs. R-ISS III median PFS months: 17.5 vs. 10 months; *p* = ns) (Fig. 4).

Notably, R-ISS I patients with a high ctDNA carried more frequently paraskeletal lesions, with an associated higher Deauville score (presence of PS lesions: 60 vs. 9.7% of patients, associated DS score = 5; 40 vs. 3.2%; *p* < 0.05), as compared to R-ISS I patients with low ctDNA; moreover, according to a multivariable logistic model, having a diffuse disease and being R-ISS III stage-defined patients were the most significantly associated factors with high ctDNA levels (odd ratios: PS DS 5 = 20.5, *p* = 0.0022; R-ISS3 = 13.3 *p* = 0.0005).

The impact on the progression of the ctDNA baseline amount was further confirmed in survival analysis, showing that patients with high ctDNA had a higher rate of progression than patients with low ctDNA (median PFS: 21 months vs not reached CI: 2.17–30.75; *p* value = 0.00183) (Supplementary Fig. 7). Having *high* ctDNA independently impacted PFS in a multivariable analysis including R-ISS III and 1q amplification (HR = 2.59, 95% CI, 1.2 to 5.7; *p* = 0.01) (Supplementary Table 6).

Thus, a specific amount of released ctDNA, equal to 12%, can distinguish MM patients with high versus those with low measured cfDNA tumor fraction in peripheral blood; moreover, a patient’s stratification, based both on the amount of ctDNA and on the R-ISS score, can accurately define patients’ prognosis.

### The higher propensity of disease spreading in patients with high cfDNA tumor fraction

One of the possible reasons for the increased release of ctDNA into peripheral blood can be the presence of multiple lesions throughout the body, sometimes also associated with spatially heterogeneous cases.

To investigate this aspect, we analyzed the whole-body PET/CT scans at baseline in patients stratified according to ctDNA levels in peripheral blood. Overall, we observed that patients with high ctDNA showed a more appreciable BM involvement, as highlighted by an overall higher BM Deauville Score (DS), even associated with higher BM SUV Max, as compared to patients with *low* ctDNA (BM DS ≥4: 11/27 vs. 15/69, *p* = 0.05). More interestingly, patients with high ctDNA levels had a higher propensity to disease spreading, particularly outside the BM. Indeed, compared to patients with low levels of cfDNA tumor fraction, they were mostly characterized by a greater incidence of focal lesions (number of focal lesions, according to ImPetUS study, ≥4: 9/27 vs. 7/69, *p* = 0.003), characterized by higher metabolic activity (focal lesions Deauville Score ≥4: 17/27 vs. 36/69, *p* = 0.03; focal lesions SUV MAX: *p* = 0.096) and frequently associated with hematogenous spread, with a higher prevalence of active extramedullary lesions (4/27 vs. 3/69, *p* = 0.09; EM lesions DS ≥4: 4/27 vs. 3/69, *p* = 0.067; EM SUV MAX: *p* = 0.008). Moreover, they also had both a higher prevalence of soft tissue infiltrations, originating from bone lesions (paraskeletal lesions) (13/27 vs. 13/69, *p* = 0.005; PM DS ≥4: 11/27 vs 10/69, *p* = 0.003; PM SUV MAX: *p* = 0.007) and a higher prevalence of hypercalcemia (calcium levels >10.5 mmol/L: 8/27 vs. 8/71, *p* = 0.036), than patients with a low ctDNA (Supplementary Table 7). By a multivariate approach we analyse the effects on PFS of this single PET/CT parameter and of the ctDNA observing that both the presence of metabolically active paraskeletal lesions (DS = 5) and of high ctDNA level impacted the patients’ outcome (Supplementary Table 8).

Hence, through the detection of ctDNA levels in peripheral blood, we have been able to identify patients characterized by a higher disease spreading, particularly associated with the presence of paraskeletal lesions.

### A CAF-mediated inflammatory state in patients with high ctDNA release

We finally focused on the possibility that specific microenvironmental conditions might have an impact on the release of different ctDNA levels into the bloodstream.

**Fig. 5:**
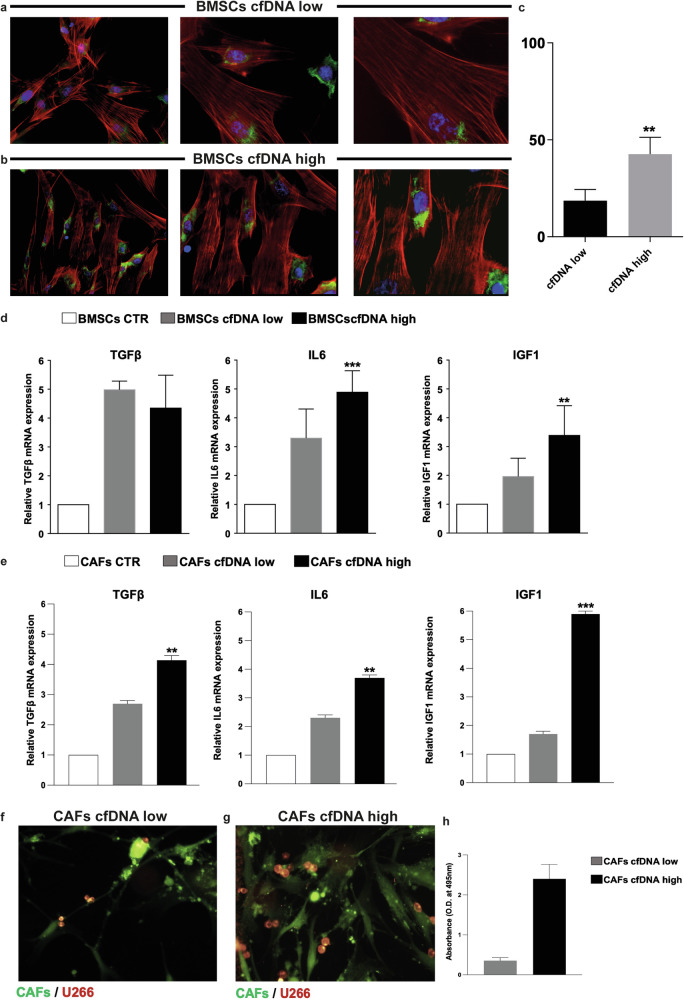
Growth factors and cytokines differential expression in bone marrow microenvironment from MM patients with low vs high cfDNA/ A CAFs-mediated inflammatory state in patients with high cfDNA release. **a**–**c** Immunofluorescence analysis, validation of increased IL-6 expression (green) in BMSCs isolated from a representative MM patient with cfDNA low (**a**) vs cfDNA high (**b**) ones at different magnifications. Cytoskeleton were labeled with phalloidin (red) and nuclei were stained using DAPI (blue). Results, expressed as mean fluorescence intensity (MFI) percentage (**c**), suggest that the presence of high levels of IL-6 in the cfDNA high cases may promote a more aggressive microenvironment. Original magnification ×20, ×40, ×63, scale bar = 25 μm. **p ≤ 0.0015. **d**, **e** qRT-PCR analysis of TGFβ, IL-6, and IGF1 mRNA expression in bone marrow stromal cells (BMSCs) (**d**) and in related cancer-associated fibroblasts (CAFs) (**e**) isolated from MM patients with cfDNA low (*n* = 8) and cfDNA high (*n* = 8), respectively. Data were expressed as mean ± SD. **p ≤ 0.0015; ***p ≤ 0.0008. **f**–**h** CAFs isolated from cfDNA high MM patients have a greater protective capacity towards MM-PCs compared to CAFs isolated from cfDNA low ones. Representative images of CAFs isolated from cfDNA low case co-cultured with MM1S (**f**) versus CAFs isolated from cfDNA high case co-cultured with MM1S (**g**). CAFs were labeled with CFSE (green), and MM1S were labeled with Dil (red). Quantification of the interaction between CAFs and MM cells, as measured by the percentage of Dil-positive MM cells that were in close proximity (within 50 μm) to CFSE-positive CAFs. The cfDNA high case showed a higher percentage of MM cells in close proximity to CAFs compared to the cfDNA low case. These results suggest that the presence of high levels of cfDNA in the cfDNA high case may promote a tighter interaction between CAFs and MM cells, potentially contributing to disease progression in MM. Original magnification 40X, scale bar 25 μm. Results were validated with an adhesion assay of MM1S (**h**) stained with Calcein-AM plated for 24 h on CAFs isolated from patients with cfDNA low (*n* = 6) and with cfDNA high (*n* = 6). Data were expressed as mean ± SD. *p < 0.002 and **p < 0.005 by Wilcoxon signed-rank test. BMSCs bone marrow stromal cells, CAFs cancer-associated fibroblasts.

To investigate this aspect, the expression level of IL-6 in BM stromal cells was observed by immunofluorescence (Fig. 5a–c). To validate the differential expression of growth factors and cytokines by BM stromal cells, we assessed the tumor microenvironment inflammatory level in patients stratified according to the amount of ctDNA (high vs. low). We observed differential mRNA expression of growth factors and cytokines (TGFβ, IL-6, and IGF1) both in BMSCs and, subsequently, in cancer-associated fibroblasts (CAFs), which might represent the main cell subpopulation responsible for inflammatory homeostasis in MM, among the two patient subgroups (Fig. 5d, e). Interestingly, BMSCs from patients with high ctDNA were also characterized by higher expression of cytokines such as CCL5 and SerpinE1, than those from patients with a low ctDNA (*p* < 0.05) (Supplementary Fig. 8a). Data were also corroborated by the analysis on high cfDNA CAFs lysates, confirming the over-expression of MIF, SerpinE1, and IL17E, strictly associated to the high tumor burden (*p* < 0.05), pinpointing CAFs as potential source of SerpinE1 (Supplementary Fig. 8b).

Finally, after cell purity characterization by α-SMA and FAP expression analysis (Supplementary Fig. 9), CAFs isolated from MM patients with high ctDNA were co-cultured with MM plasma cells, showing a greater protective capacity toward MM-PCs, than CAFs isolated from patients with low ctDNA (Fig. 5f, g). The strict interaction between CAFs and MM-PCs was confirmed by an adhesion assay, which highlighted the tight connection between these two cell types (Fig. 5h).

Overall, based on gene expression and adhesion assay data, we deemed soluble- and cell-mediated interactions between MM-PCs and CAFs to be boosted in high ctDNA patients.

### The cfDNA tumor fraction is highly informative, particularly when the BM might be not fully representative of the disease

In the recent years, increasing evidence has suggested that cfDNA can be considered a non-invasive source of genomic information, particularly in the case of hypocellular BM aspirates, which might prevent the attainment of the information needed.

Indeed, for a subgroup of 22 out of 160 patients (13.7%), FISH analyses were not feasible due to low plasma cell enrichment (plasma cell number <0.08 × 10^6^). In 9 out of 22 patients (40.9%), we were able to infer the CNV profile by ULP-WGS on both gDNA and cfDNA, whereas in the remaining 13 (59.6%), only the gDNA was informative and rich enough to permit the CNV calling.

Interestingly, cfDNA can also be used in SPC cases, whose BM aspirates from the iliac crest are commonly not informative enough and/or the cellularity is modest. Indeed, CNVs were defined from cfDNA analysis in 4 out of 7 SPC cases (57.1%), potentially avoiding the SPC biopsy. (Supplementary Fig. 10).

Therefore, in cases where BM aspirate is not informative, cfDNA can provide suitable markers for disease monitoring while being less invasive than BM aspirate. Moreover, in the case of cfDNA analysis failure (e.g., when the tumor fraction is below the limit of detection <3%), the CNV profile can be detected in gDNA obtained from BM aspirates by ULP-WGS, as this approach requires less material to be informative than the commonly employed FISH panel.

## Discussion

In recent years, increasing attention has been devoted to the possible introduction of less invasive methods for MM disease characterization and prognostication, such as liquid biopsy, that could be integrated within the current gold standard methods (e.g., FISH on bone marrow) and, possibly, for minimal residual disease monitoring. Emerging data suggest that a less invasive biological source, such as cfDNA, could be suitable, particularly in those patients where BM aspirate cellularity can be poor, or to effectively guide decision-making at the bedside [31].

Previous works have reported that a high amount of total cfDNA in RRMM patients was correlated with inferior PFS [20]. Our data on NDMM demonstrate that only the tumor portion of total cfDNA is effectively relevant for patients’ outcomes and can quantitatively mimic the tumor clone’s features. Indeed, the cfDNA tumor fraction in peripheral blood, also referred to as ctDNA, is well correlated with the total BM plasma cell amount, as measured both by morphology and by b2M. Conversely, the total amount of cfDNA cannot reflect BM disease burden, as it can be influenced by a plethora of external factors, not strictly related to myeloma disease, that have not been considered in our analysis (concomitant cardiac failures, stress level, etc.) [32]. Moreover, the two-site BM-PB genomic analysis demonstrates that cfDNA can effectively be a proxy of the tumor molecular fingerprint, derived from lesions with regionally different distributions. Indeed, in most patients, the cfDNA CNV profile is superimposable to that derived from BM, at least for clonal alterations, further supporting its advantageous role in reflecting the BM neoplastic clone, both quantitatively and qualitatively.

This evidence led us to further investigate the role of cfDNA under therapy, when the tumor burden in BM tends to shrink. To this aim, a subgroup of 22 patients, upfront treated with triplets including anti-CD38 mAb, was monitored by a trimodal approach (cfDNA-BM-PET/CT), up to the 18th month under lenalidomide maintenance, demonstrating that in most cases, the ctDNA released into the bloodstream reflected the amount of residual BM disease, and confirming the PET scan results.

Due to the sequencing approach employed, we have been unable to confidently infer a possible presence of spatial heterogeneity, neither to effectively measure MRD by liquid biopsy. Recent studies have highlighted the possibility of using more performing methods, such as phased variant sequencing [31], that could be readily adopted in the future to characterize both CNAs and SNVs at higher sensitivity moving forward from a punctual to a kinetic method of MRD determination, through liquid biopsy. Our data support the possible upcoming development of a new sequential MRD strategy, that employs peripheral blood to assess the disease dynamics, unless a negative result is obtained, performing BM aspirates just to confirm the disease clearence [33]. In this setting, also the detection of M-proteins by mass spectrometry seems currently to be an advantageous liquid biopsy method to monitor MRD in peripheral blood. However, its applicability is still limited to dedicated laboratories and is not yet standardized [34].

**Fig. 6:**
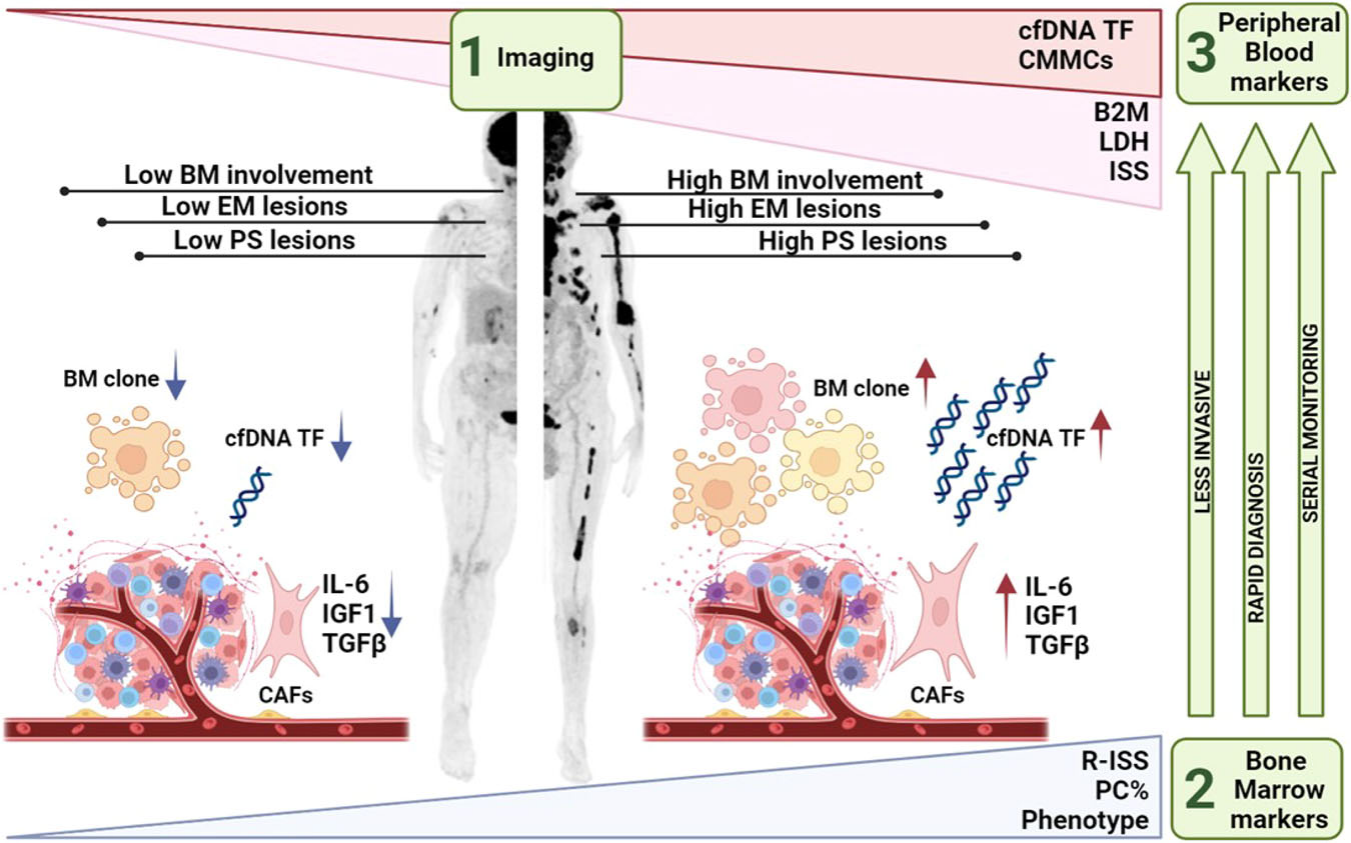
MM patients’ risk definition by a trimodality approach. To best characterize the risk of progression of MM patients, the disease must be studied at three levels: in the bone marrow (R-ISS3 and cytogenetics), in the whole body (paraskeletal and extramedullary lesions), and in peripheral blood (ctDNA), enabling to face with the multilayer disease heterogeneity.

Interestingly, we showed that the definition of high ctDNA release, based on a 12% cut-off, has a clinical impact on patients’ outcomes. However, the prognostic impact of a single marker, though significant, can just partially justify the observed outcome. Indeed, recent evidences have suggested that both an increasing number and a synergistic effect of different interacting parameters can increase the sensitivity and reliability of risk determination [35]. Here we show that the integration of ctDNA in patients’ risk stratification scoring systems allows a more refined definition of the individual risk of progression. This was particularly evident for R-ISS I low-risk patients, whose risk definition was more accurately defined by considering also the measure of released ctDNA amount. Moreover, the logistic model suggested that not only R-ISS III significantly correlates with ctDNA, but also the presence of paraskeletal lesions (DS = 5), suggesting that ctDNA, through a less invasive and relatively cheap method, might represent a potential warning of both aggressive and diffused disease. Although this study was not supported by TAC-guided biopsies of single lesions, these findings can add a significant contribution to the role of ctDNA as a proxy of the whole disease. These results overall suggest that the risk of progression of MM patients is best characterized by studying the disease at three levels (Fig. 6): within the BM (R-ISS3 and cytogenetics), at the whole body level (PS and EM lesions), and within peripheral blood (ctDNA), enabling the assessment of a multilayer disease heterogeneity. Indeed, the multivariate analysis demonstrates that having both metabolically active paraskeletal lesions and high ctDNA impacted PFS; however, data from ctDNA have the advantage to be more easily available, as compared to those from PET/CT test, as well as less invasive for the patient.

Overall, the different levels of released ctDNA might be the consequence of a different turnover of plasma cells located in differently distributed focal lesions, where either a high or a low local inflammatory microenvironment might condition plasma cells death. On top of that, our data indicate that BMSCs and CAFs exhibit a more inflammatory, tumor-promoting state in high ctDNA MM patients than in *low* ctDNA patients. BMSCs contribute to the pro-inflammatory tumor microenvironment by expressing key factors such as IL-1β, TNFα, LIF, and COX2, which support MM proliferation [36]. CAFs assert their pivotal role within this vicious cycle, actively shaping an inflamed neoplastic niche where IL-6, IL-11, LIF, and CXCL5 orchestrate an inflammatory phenotype that fuels cancer progression [37]. Future studies will focus on further characterizing this subtype and its potential as a therapeutic target in the tumor microenvironment. In particular, CAFs from high ctDNA patients showed increased protection of MM cells and more frequent direct interactions [38, 39]. This supportive microenvironment likely contributes to aggressive disease progression in high ctDNA patients, showing a tight interaction between CAFs and MM cells, possibly suggesting an active role of cfDNA in immune system self-tolerance breakage, followed by the establishment of a tumor-promoting microenvironment [23]. An essential factor mediating the crosstalk between CAFs and MM cells may be SerpinE1, also known as plasminogen activator inhibitor-1 (PAI-1) [40]. SerpinE1 is an inhibitor of urokinase plasminogen activator (uPA) and tissue plasminogen activator (tPA), which are implicated in cancer progression and metastasis [41]. Recent studies have found that MM CAFs secrete high levels of SerpinE1, which promotes MM cell migration, drug resistance, and angiogenesis. SerpinE1 expression in CAFs is driven by inflammatory cytokines such as TGFβ and IL-6, which were upregulated in BMSCs from high ctDNA patients in this study, paving the way for crucial therapeutic implications [42]. Therefore, CAFs in the high ctDNA microenvironment likely express high levels of SerpinE1, which could, in turn, facilitate MM cell growth, survival, and dissemination [43]. SerpinE1 can also induce DNA damage and apoptosis resistance in cancer cells. The cytotoxic DNA damage caused by SerpinE1 may directly contribute to the release of tumor DNA into the circulation, driving the high ctDNA levels associated with poor prognosis [38, 44]. Further analyses of SerpinE1 expression and activity in primary CAFs from low vs. high ctDNA MM patients could help validate the role of this pathway in the aggressive high ctDNA phenotype [45]. Inhibition of SerpinE1 in coculture models may also reveal its necessity for MM cell ctDNA release. Targeting the SerpinE1 axis could represent a novel strategy to disrupt the tumor-promoting microenvironment and reduce ctDNA levels in high-risk MM as already demonstrated in other cancer models [46]. Recent studies have found that MM CAFs secrete high levels of SerpinE1, which promotes MM cell migration, drug resistance, and angiogenesis. SerpinE1 expression in CAFs is driven by inflammatory cytokines such as TGFβ and IL-6, which were upregulated in BMSCs from *high* ctDNA patients in this study, paving the way for crucial therapeutic implications [42]. Therefore, CAFs in the *high* ctDNA microenvironment likely express high levels of SerpinE1, which could, in turn, facilitate MM cell growth, survival, and dissemination [43]. SerpinE1 can also induce DNA damage and apoptosis resistance in cancer cells. The cytotoxic DNA damage caused by SerpinE1 may directly contribute to the release of tumor DNA into the circulation, driving the high ctDNA levels associated with poor prognosis [43, 44]. Further analyses of SerpinE1 expression and activity in primary CAFs from *low vs*. *high* ctDNA MM patients could help validate the role of this pathway in the aggressive *high* ctDNA phenotype [45]. Inhibition of SerpinE1 in coculture models may also reveal its necessity for MM cell ctDNA release. Targeting the SerpinE1 axis could represent a novel strategy to disrupt the tumor-promoting microenvironment and reduce ctDNA levels in high-risk MM as already demonstrated in other cancer models [46].

In summary, we provided evidence showing that ctDNA can be a reliable and low invasive marker for disease characterization. Moreover, if evaluated along with both PET/CT and BM, it can refine and/or better define the patients’ risk. In addition, the analysis of ctDNA allows - with a single assay - both to capture the patient’s whole genomic profile and to quantify the tumor fraction, which is finally correlated with patients’ risk of progression. To conclude, this work highlights the relevance of cfDNA in myeloma and further paves the way for the inclusion of peripheral markers in patient risk stratification.

## Availability of data and materials

the datasets used and/or analysed during the current study are available from the corresponding author on reasonable request.

## Supplementary information

Supplementary

## Data Availability

The datasets used and/or analysed during the current study are available from the corresponding author on reasonable request.

## References

[1] Kumar SK, Rajkumar SV, Dispenzieri A, Lacy MQ, Hayman SR, Buadi FK, et al. Improved survival in multiple myeloma and the impact of novel therapies. Blood. 2008;111:2516–20. 17975015 10.1182/blood-2007-10-116129 PMC2254544

[2] Rodriguez-Otero P, Paiva B, San-Miguel JF. Roadmap to cure multiple myeloma. Cancer Treat Rev. 2021;100:102284. 34597912 10.1016/j.ctrv.2021.102284

[3] Neri P, Bahlis NJ, Lonial S. New strategies in multiple myeloma: immunotherapy as a novel approach to treat patients with multiple myeloma. Clin Cancer Res. 2016;22:5959–65. 27797968 10.1158/1078-0432.CCR-16-0184

[4] Mohan M, Hari P, Dhakal B. Immunotherapy in multiple myeloma-time for a second major paradigm shift. JCO Oncol Pract. 2021;17:405–13. 34003675 10.1200/OP.21.00032

[5] Romano A, Palumbo GA, Parrinello NL, Conticello C, Martello M, Terragna C. Minimal residual disease assessment within the bone marrow of multiple myeloma: a review of caveats, clinical significance and future perspectives. Front Oncol. 2019;9:699. 31482061 10.3389/fonc.2019.00699 PMC6710454

[6] Paiva B, van Dongen JJ, Orfao A. New criteria for response assessment: role of minimal residual disease in multiple myeloma. Blood. 2015;125:3059–68. 25838346 10.1182/blood-2014-11-568907 PMC4513329

[7] Zamagni E, Nanni C, Mancuso K, Tacchetti P, Pezzi A, Pantani L, et al. PET/CT improves the definition of complete response and allows to detect otherwise unidentifiable skeletal progression in multiple myeloma. Clin Cancer Res. 2015;21:4384–90. 26078390 10.1158/1078-0432.CCR-15-0396

[8] Latifoltojar A, Boyd K, Riddell A, Kaiser M, Messiou C. Characterizing spatial heterogeneity of multiple myeloma in high resolution by whole-body magnetic resonance imaging: towards macro-phenotype driven patient management. Magn Reson Imaging. 2021;75:60–64. 33075451 10.1016/j.mri.2020.10.005

[9] Rasche L, Angtuaco EJ, Alpe TL, Gershner GH, McDonald JE, Samant RS, et al. The presence of large focal lesions is a strong independent prognostic factor in multiple myeloma. Blood. 2018;132:59–66. 29784643 10.1182/blood-2018-04-842880 PMC6034645

[10] Rasche L, Chavan SS, Stephens OW, Patel PH, Tytarenko R, Ashby C, et al. Spatial genomic heterogeneity in multiple myeloma revealed by multi-region sequencing. Nat Commun. 2017;8:268. 28814763 10.1038/s41467-017-00296-y PMC5559527

[11] Rasche L, Schinke C, Maura F, Bauer MA, Ashby C, Deshpande S, et al. The spatio-temporal evolution of multiple myeloma from baseline to relapse-refractory states. Nat Commun. 2022;13:4517. 35922426 10.1038/s41467-022-32145-y PMC9349320

[12] Moreau P, Hulin C, Perrot A, Arnulf B, Belhadj K, Benboubker L, et al. Maintenance with daratumumab or observation following treatment with bortezomib, thalidomide, and dexamethasone with or without daratumumab and autologous stem-cell transplant in patients with newly diagnosed multiple myeloma (CASSIOPEIA): an open-label, randomised, phase 3 trial. Lancet Oncol. 2021;22:1378–90. 34529931 10.1016/S1470-2045(21)00428-9

[13] Yee AJ, Raje N. Minimal residual disease in multiple myeloma: why, when, where. Hematol Am Soc Hematol Educ Program. 2021;2021:37–45. 10.1182/hematology.2021000230 PMC8791109 34889430

[14] Lone SN, Nisar S, Masoodi T, Singh M, Rizwan A, Hashem S, et al. Liquid biopsy: a step closer to transform diagnosis, prognosis and future of cancer treatments. Mol Cancer. 2022;21:79. 35303879 10.1186/s12943-022-01543-7 PMC8932066

[15] Bertamini L, Oliva S, Rota-Scalabrini D, Paris L, More S, Corradini P, et al. High levels of circulating tumor plasma cells as a key hallmark of aggressive disease in transplant-eligible patients with newly diagnosed multiple myeloma. J Clin Oncol. 2022;40:3120–31. 35666982 10.1200/JCO.21.01393

[16] Faict S, Muller J, De Veirman K, De Bruyne E, Maes K, Vrancken L, et al. Exosomes play a role in multiple myeloma bone disease and tumor development by targeting osteoclasts and osteoblasts. Blood Cancer J. 2018;8:105. 30409995 10.1038/s41408-018-0139-7 PMC6224554

[17] Manier S, Liu CJ, Avet-Loiseau H, Park J, Shi J, Campigotto F, et al. Prognostic role of circulating exosomal miRNAs in multiple myeloma. Blood. 2017;129:2429–36. 28213378 10.1182/blood-2016-09-742296 PMC5409448

[18] Corcoran RB, Chabner BA. Application of cell-free DNA analysis to cancer treatment. N Engl J Med. 2018;379:1754–65. 30380390 10.1056/NEJMra1706174

[19] Stejskal P, Goodarzi H, Srovnal J, Hajduch M, van ‘t Veer LJ, Magbanua MJM. Circulating tumor nucleic acids: biology, release mechanisms, and clinical relevance. Mol Cancer. 2023;22:15. 36681803 10.1186/s12943-022-01710-w PMC9862574

[20] Waldschmidt JM, Yee AJ, Vijaykumar T, Pinto RA, Frede J, Anand P, et al. Cell-free DNA for the detection of emerging treatment failure in relapsed/ refractory multiple myeloma. Leukemia. 2022;36:1078–87. 35027656 10.1038/s41375-021-01492-y PMC8983453

[21] Thakral D, Das N, Basnal A, Gupta R. Cell-free DNA for genomic profiling and minimal residual disease monitoring in Myeloma- are we there yet? Am J Blood Res. 2020;10:26–45. 32685257 PMC7364270

[22] Manier S, Park J, Capelletti M, Bustoros M, Freeman SS, Ha G, et al. Whole-exome sequencing of cell-free DNA and circulating tumor cells in multiple myeloma. Nat Commun. 2018;9:1691. 29703982 10.1038/s41467-018-04001-5 PMC5923255

[23] Korabecna M, Zinkova A, Brynychova I, Chylikova B, Prikryl P, Sedova L, et al. Cell-free DNA in plasma as an essential immune system regulator. Sci Rep. 2020;10:17478. 33060738 10.1038/s41598-020-74288-2 PMC7566599

[24] Lauer EM, Mutter J, Scherer F. Circulating tumor DNA in B-cell lymphoma: technical advances, clinical applications, and perspectives for translational research. Leukemia. 2022;36:2151–64. 35701522 10.1038/s41375-022-01618-w PMC9417989

[25] Bohers E, Viailly PJ, Becker S, Marchand V, Ruminy P, Maingonnat C, et al. Non-invasive monitoring of diffuse large B-cell lymphoma by cell-free DNA high-throughput targeted sequencing: analysis of a prospective cohort. Blood Cancer J. 2018;8:74. 30069017 10.1038/s41408-018-0111-6 PMC6070497

[26] Greipp PR, San Miguel J, Durie BG, Crowley JJ, Barlogie B, Blade J, et al. International staging system for multiple myeloma. J Clin Oncol. 2005;23:3412–20. 15809451 10.1200/JCO.2005.04.242

[27] Palumbo A, Avet-Loiseau H, Oliva S, Lokhorst HM, Goldschmidt H, Rosinol L, et al. Revised international staging system for multiple myeloma: a report from International Myeloma Working Group. J Clin Oncol. 2015;33:2863–9. 26240224 10.1200/JCO.2015.61.2267 PMC4846284

[28] Ewels P, Magnusson M, Lundin S, Kaller M. MultiQC: summarize analysis results for multiple tools and samples in a single report. Bioinformatics. 2016;32:3047–8. 27312411 10.1093/bioinformatics/btw354 PMC5039924

[29] Adalsteinss on VA, Ha G, Freeman SS, Choudhury AD, Stover DG, Parsons HA, et al. Scalable whole-exome sequencing of cell-free DNA reveals high concordance with metastatic tumors. Nat Commun. 2017;8:1324. 29109393 10.1038/s41467-017-00965-y PMC5673918

[30] Mazzocchetti G, Poletti A, Solli V, Borsi E, Martello M, Vigliotta I, et al. BoBafit: a copy number clustering tool designed to refit and recalibrate the baseline region of tumors’ profiles. Comput Struct Biotechnol J. 2022;20:3718–28. 35891790 10.1016/j.csbj.2022.06.062 PMC9294200

[31] Kurtz DM, Soo J, Co Ting Keh L, Alig S, Chabon JJ, Sworder BJ, et al. Enhanced detection of minimal residual disease by targeted sequencing of phased variants in circulating tumor DNA. Nat Biotechnol. 2021;39:1537–47. 34294911 10.1038/s41587-021-00981-w PMC8678141

[32] Grabuschnig S, Bronkhorst AJ, Holdenrieder S, Rosales Rodriguez I, Schliep KP, Schwendenwein D, et al. Putative origins of cell-free DNA in humans: a review of active and passive nucleic acid release mechanisms. Int J Mol Sci. 2020;21:8062. 10.3390/ijms21218062 PMC7662960 33137955

[33] Kubicki T, Derman BA, Dytfeld D, Jakubowiak AJ. Measurable residual disease in peripheral blood in myeloma: dream or reality. Curr Opin Oncol. 2023;35:574–80 10.1097/CCO.0000000000000987 37621165

[34] Chapman JR, Thoren KL. Tracking of low disease burden in multiple myeloma: using mass spectrometry assays in peripheral blood. Best Pr Res Clin Haematol. 2020;33:101142. 10.1016/j.beha.2020.101142 32139008

[35] Zviran A, Schulman RC, Shah M, Hill STK, Deochand S, Khamnei CC, et al. Genome-wide cell-free DNA mutational integration enables ultra-sensitive cancer monitoring. Nat Med. 2020;26:1114–24. 32483360 10.1038/s41591-020-0915-3 PMC8108131

[36] Kuang C, Zhu Y, Guan Y, Xia J, Ouyang J, Liu G, et al. COX2 confers bone marrow stromal cells to promoting TNFα/TNFR1β-mediated myeloma cell growth and adhesion. Cell Oncol. 2021;44:643–59. 10.1007/s13402-021-00590-4 PMC12980671 33646559

[37] Yang D, Liu J, Qian H, Zhuang Q. Cancer-associated fibroblasts: from basic science to anticancer therapy. Exp Mol Med. 2023;55:1322–32. 37394578 10.1038/s12276-023-01013-0 PMC10394065

[38] Mao X, Xu J, Wang W, Liang C, Hua J, Liu J, et al. Crosstalk between cancer-associated fibroblasts and immune cells in the tumor microenvironment: new findings and future perspectives. Mol Cancer. 2021;20:131. 34635121 10.1186/s12943-021-01428-1 PMC8504100

[39] Saltarella I, Lamanuzzi A, Desantis V, Di Marzo L, Melaccio A, Curci P, et al. Myeloma cells regulate miRNA transfer from fibroblast-derived exosomes by expression of lncRNAs. J Pathol. 2022;256:402–13. 34919276 10.1002/path.5852

[40] Purdue MP, Lan Q, Menashe I, Zheng T, Zhang Y, Yeager M, et al. Variation in innate immunity genes and risk of multiple myeloma. Hematol Oncol. 2011;29:42–46. 20658475 10.1002/hon.954 PMC2980579

[41] Xu S, De Veirman K, De Becker A, Vanderkerken K, Van Riet I. Mesenchymal stem cells in multiple myeloma: a therapeutical tool or target? Leukemia. 2018;32:1500–14. 29535427 10.1038/s41375-018-0061-9 PMC6035148

[42] Sakemura R, Hefazi M, Siegler EL, Cox MJ, Larson DP, Hansen MJ, et al. Targeting cancer-associated fibroblasts in the bone marrow prevents resistance to CART-cell therapy in multiple myeloma. Blood. 2022;139:3708–21. 35090171 10.1182/blood.2021012811 PMC11290597

[43] Pan JX, Qu F, Wang FF, Xu J, Mu LS, et al. Aberrant SERPINE1 DNA methylation is involved in carboplatin induced epithelial-mesenchymal transition in epithelial ovarian cancer. Arch Gynecol Obstet. 2017;296:1145–52. 28975405 10.1007/s00404-017-4547-x

[44] Pavon MA, Arroyo-Solera I, Tellez-Gabriel M, Leon X, Viros D, Lopez M, et al. Enhanced cell migration and apoptosis resistance may underlie the association between high SERPINE1 expression and poor outcome in head and neck carcinoma patients. Oncotarget. 2015;6:29016–33. 26359694 10.18632/oncotarget.5032 PMC4745708

[45] Ferrucci A, Moschetta M, Frassanito MA, Berardi S, Catacchio I, Ria R, et al. A HGF/cMET autocrine loop is operative in multiple myeloma bone marrow endothelial cells and may represent a novel therapeutic target. Clin Cancer Res. 2014;20:5796–807. 25212607 10.1158/1078-0432.CCR-14-0847

[46] McCann JV, Xiao L, Kim DJ, Khan OF, Kowalski PS, Anderson DG, et al. Endothelial miR-30c suppresses tumor growth via inhibition of TGF-beta-induced Serpine1. J Clin Invest. 2019;129:1654–70. 30855280 10.1172/JCI123106 PMC6436861

