# Supplementary material for: High level of circulating cell-free tumor DNA at diagnosis correlates with disease spreading and defines multiple myeloma patients with poor prognosis
Source: Blood Cancer J. 2024 Nov 28;14(1):208. doi: 10.1038/s41408-024-01185-6 (PMC11605000; doi:10.1038/s41408-024-01185-6)
Supplement: Supplementary file 1 — Supplementary [file 41408_2024_1185_MOESM1_ESM.docx]

**SUPPLEMENTARY MATERIALS AND METHODS**

Complete Materials and Methods section have been reported here in the Supplementary file.

**Participant enrolment and clinical sample collection**

A total of 162 newly diagnosed multiple myeloma patients were enrolled in this study, for whom peripheral blood and bone marrow samples were available. In addition, 7 solitary plasmacytoma [SPC] and 7 smouldering myeloma [SMM] were also included to investigate the role of cfDNA in these clinical settings. Informed consent for treatment and sample procurement by the Declaration of Helsinki was obtained for all cases included in the study (AIRC IG2018 "StreaMMing” project approved by ethical committee EC n. 167/2019/Sper/AOUBO). The cohort of MM patients was representative of an overall MM patient population, as shown in **Suppl. Tab. 1**, reporting the distribution of cytogenetic alterations, ISS and R-ISS stages and baseline clinical characteristics. All the translocations (t4;14, t11;14, t14;16, t6;14 and t14;20) and del17p, del1p32 and 1q21+ were assessed by FISH analyses (Vysis LSI Probes, Abbott Molecular).

SMM patients were routinely monitored but did not receive any treatments. Conversely, MM patients were mostly upfront treated with 3 or 4 drug regimens including CD38 mAb (up to 60% achieved at least a very good partial response). After a single or double ASCT, they received a consolidation and then a maintenance treatment (**Suppl. Tab. 2**). The project started the enrollment in early 2019 and the median follow-up of the patient cohort was 36 months.

A specific biological sample biobanking was planned (**Fig. 1**), which included a BM sample at diagnosis, BM sample every 6 months, and PB samples monthly. PB was collected in EDTA tubes and processed for plasma isolation within 4 hours after collection avoiding genomic DNA contamination. According to this schema, a total of 271 PB samples were collected monthly for cfDNA analysis in a subset of 22 patients. In all patients, plasma cells were isolated from BM samples by immunomagnetic anti-CD138 bead method (Miltenyi Biotec, Germany) at diagnosis as previously described^28^ to identify the immunoglobulin and light chain clonotypes and monitored by NGS on total BM, collected under treatment (Lymphotrack® Dx, Invivoscribe, San Diego, CA). Cell-free DNA from plasma and gDNA from BM samples were isolated using dedicated kits (Promega). cfDNA quantity and quality was checked at Tapestation by using the genomic DNA kit which permit to identify any possible genomic DNA contamination. Whenever ctDNA is mentioned in the text, reference is made to cfDNA tumor fraction.

**Whole body imaging low dose PET-CT**

All radiographic imaging was performed as part of standard clinical care. By study design, 96/162 patients were studied at baseline with 18F-FDG PET-CT. PET/CT was also repeated after ASCT and during post-ASCT follow-up approximately once a year; its evaluation after induction treatment was not mandatory, but at the physician’s discretion. All PET/CT scans were acquired according to the European Association of Nuclear Medicine PET procedures guidelines for FDG studies^29^. Patients were injected intravenously with 2-3 MBq/Kg of 18F-FDG, with an expected uptake time of approximately 60+/- 10 min in all patients; 6 hours of fasting was needed for all patients before injection. Antidiabetic therapy was discontinued on the day of the examination. Images were acquired on a 3D tomograph (Discovery STE; GE) for 2 min for each bed position. Low-dose CT (120 kV, 20 mA) was used both for attenuation correction and to provide an anatomic map. Whole-body field of view acquisition (including skull, superior limbs, femurs, and proximal tibias) was carried out and reported following the Italian Myeloma Criteria for PET Use (IMPeTUs; elaborated within the EMN02/HOVON95 trial^30^). These criteria are a comprehensive evaluation including various semiquantitative parameters (SUVmax, PET FLs, CT lytic lesions, fracture lesions, PMD/EMD disease) and they are based on the Deauville five point scale (D5-PS) exactly as lymphomas^31^. In particular, bone marrow metabolic state (BM), focal lesions with or without osteolytic characteristics (FLs; number and metabolic state), paraskeletal lesions (site, number and metabolic rate), consisting of soft-tissue masses arising from bone lesions, and extramedullary lesions (site, number, and metabolic state) consisting of extraosseous tissue, involving only soft tissues, were checked and reported. The SUVmax of the hottest FL, the hottest paraskeletal and extramedullary lesion, and diffuse pathological bone marrow (BM) involvement were reported. BM was interpreted as positive for myeloma in 18F-FDG scan if DS≥4; all the areas of focal uptake were interpreted as visually positive unless they were at sites of known accumulation (kidneys and bladder, gastrointestinal tract, and skeletal areas showing symmetric joint uptake, especially within the shoulder girdle) and even in absence of a clear anatomic correlation. Furthermore, semiquantitative parameters, such as liver and mediastinal blood pool SUVmax and BM SUVmax of the hottest lesion per macro area, were annotated and used to reinforce visual analysis interpretation, especially in borderline cases (**Suppl. Tab. 3**).

**Ultra Low Pass Whole Genome Sequencing**

Ultra low-pass WGS was performed both on cfDNA from plasma and on gDNA derived from BM to identify the gDNA and cfDNA tumor fractions and the grade of similarity between the two tissues. A total of 1 and 10 ng of cfDNA and tumor gDNA, respectively, were used for library preparation (Takara), and a 150-bp single read sequencing strategy to a median sequencing depth of 0,47X (interquartile range: 0,26X-0,72X) was employed on a NextSeq 500 system (Illumina, CA, USA).

**Cell purification and cultures**

BM mononuclear cells (BMMCs) were isolated from heparinized BM aspirates via Ficoll–Hypaque (Sigma, St. Louis, MO, USA) gradient separation. Bone marrow stromal cells (BMSCs) were obtained after the adhesion of BMMCs to polystyrene flasks and cultured in DMEM (Euroclone, Milan, Italy) with the addition of 1% penicillin/streptomycin (Euroclone) and 10% fetal bovine serum (FBS; Sigma). Cancer-associated fibroblasts (CAFs) were isolated from BMSCs through D7-FIB-conjugated (anti-fibroblasts) microbeads (Miltenyi, Auburn, CA, USA) and cultured with DMEM (Euroclone) supplemented with 1% penicillin/streptomycin (Euroclone) and 20% FBS (Sigma Aldrich). Cell purity (≥95%) was assessed by flow cytometry by analyzing the expression of αSMA (FITC) and FAP (APC) proteins on FACSCanto II cytofluorimeter (Becton Dickinson-BD, San Jose, CA, USA)^32^. MM1S and U266 cell lines were obtained from the American Type Culture Collection (ATCC). Human Bone Marrow Stromal Cells line was obtained from Lonza (Cat. #2M-302).

**Real-Time Quantitative RT-PCR (RT-qPCR)**

Total RNA was isolated from 1×106 BMSCs and CAFs using the RNeasy Mini kit (Qiagen, Milano, Italy) and reverse-transcribed into total cDNA with the iScript cDNA Synthesis kit (Bio-Rad, Hercules, CA, USA) according to the manufacturer’s instructions. Gene expression was analyzed using SsoAdvanced Universal Probes Supermix (Bio-Rad) and specific TaqMan assays (Gapdh: Hs02758991_g1, TGFβ: Hs00998133_m1, IL6: Hs00985639_m1, IGF1: Hs01547656_m1). Relative gene expression was normalized to GAPDH as an endogenous control, and the relative fold changes were measured using the 2-∆∆Ct method.

**Immunofluorescence Staining of IL-6 in MM BMSCs**

BMSCs (1x10^5^) derived from MM patients were processed and isolated according to established protocols. Next, the cells were cultured onto microscope slides suitable for microscopy until they reached the desired confluence. For the fixation step, cells were treated with 4% paraformaldehyde for 15 minutes at room temperature, followed by three rinses with phosphate-buffered saline (PBS, Euroclone). After fixation, the cells were permeabilized using 0.1% Triton X-100 in PBS, maintained for 15 minutes at room temperature, and washed thrice with PBS. To block nonspecific binding sites, a one-hour incubation at room temperature with a blocking buffer containing 5% bovine serum albumin (BSA) in PBS was carried out in a humidified chamber. Subsequently, the cells were incubated overnight at 4°C in a humidified chamber with the primary anti-IL-6 antibody at a dilution recommended by the supplier (OTI3G9 clone, PMID: 28467474). Following the primary antibody incubation, the cells were washed three times with PBS to remove any unbound antibodies. The secondary antibody, conjugated with a suitable fluorescence dye and matching the primary antibody's host species, was applied at the supplier-recommended dilution. Cells were incubated with this secondary antibody for 1 hour at room temperature in a dark environment. After secondary antibody incubation, nuclear staining was performed using DAPI for a few minutes, followed by a thorough wash with PBS, repeated thrice. The samples were then mounted using a fluorescent mounting medium and left to dry. Visualization was conducted using a fluorescence microscope equipped with the correct filter sets for the utilized fluorophores. Image acquisition and fluorescence intensity analysis were carried out using appropriate imaging software, adhering to standard protocols to maintain integrity and consistency in the results. For the control, a set of samples was solely incubated with the secondary antibody to assess the specificity of the staining process. Safety guidelines were strictly followed throughout the procedure, especially while handling human samples and chemicals. A total of 1×10^5^ CAFs labeled with CFSE (Thermo Fisher Scientific), were cocultured on fibronectin-coated chamber slides (LabTek) with 1×105 U266 cells (labeled with Vybrant™ DiI Cell-Labeling Solution, Thermo Fisher Scientific) for 72 hours. Images were obtained with an Olympus fluorescence microscope (Olympus Italia, Rozzano, Italy).

**Adhesion assay**

MM1S were stained with Calcein AM for 1h, and then plated in triplicate in 96-well on CAFs isolated from cfDNAlow and cfDNAhigh patients (ratio= MM1S:CAFs, 2:1). After 24 hours, non-adherent cells were washed away and the rate of adherent cells was evaluated reading fluorescence at 495 nm by VICTOR™ X3 Multilabel Plate Reader (PerkinElmer Inc., Waltham, MA, USA).

**Human Cytokine Array**

BM plasma and cell lysates (300 µg) from fresh purified CAFs were analyzed using the Proteome Profiler Human Cytokine Array Kit (R&D System)) according to the manufacturer's instructions. Densitometric quantification of the resulting membranes was performed using Kodak Molecular Imaging Software (Eastman Kodak Co) and the average pixel density of each protein was normalized to reference spots.

**Bioinformatic and statistical analyses**

FASTQ files were obtained and analyzed using MultiQC^33^ to evaluate experimental metrics. BAM files were generated by applying GATK best practices for data preprocessing, including read mapping to the reference genome (version hg19), marking duplicates and base quality score recalibration. BAM files were then processed through IchorCNA^34^, which uses a probabilistic model, implemented as a hidden Markov model (HMM), to simultaneously segment the genome, predict large-scale copy number alterations, and estimate the tumor fraction of a ULP-WGS profile. cfDNA and gDNA comparison was feasible in a subgroup of 62 patients for a total of 162 MM samples at baseline. Samples were selected based on their tumor fraction (>3%) and MAD (MAD < 0.20) both estimated by the ichorCNA tool. The MAD cutoff was estimated based on a manual comparison of the profiles obtained with ichorCNA and SNParray. CN values were estimated from the average signal and corrected for the sample ploidy obtained by BoBafit^35^. The cfDNA cutoff predictive for prognosis in terms of PFS and OS has been determined through a receiving operating characteristics (ROC) curve. Comparisons between patient groups were performed using Pearson’s χ2 test or Fisher’s exact test, as appropriate, for categorical data and the Kruskal Wallis test for continuous data. The %CV (standard deviation/mean*100) was also used to assess variability. Survival analyses have been defined according to Kaplan-Meier survival curves.

**SUPPLEMENTARY**

**FIGURES AND TABLES**

**Supplementary Table 1: Patients’ baseline clinical and genomic characteristics.**

**Supplementary Table 2: Therapeutic regimens.**

**Supplementary Table 3: Patients’ baseline PET characteristics**


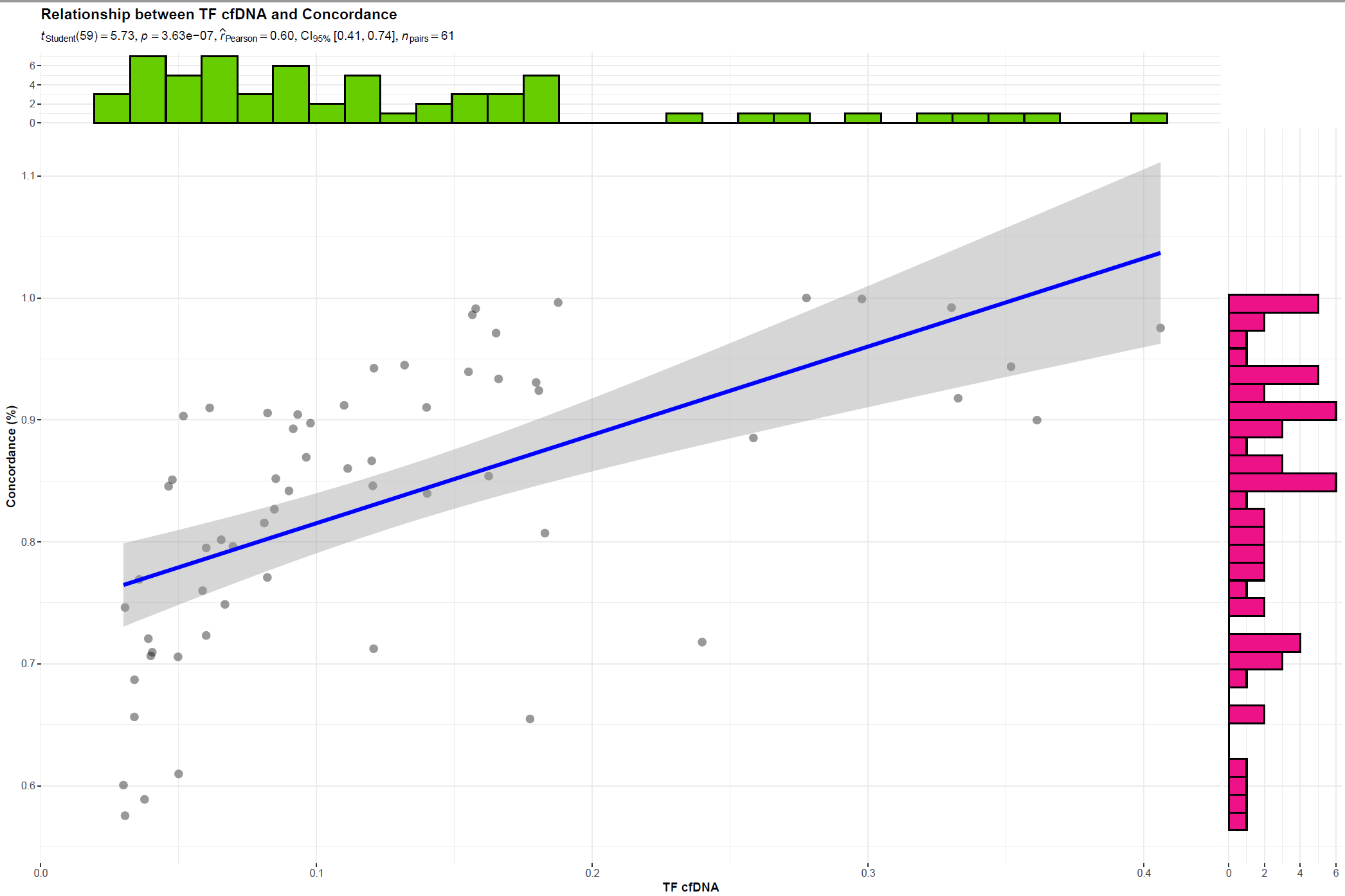


**Supplementary figure 1: The clustering efficiency is a function of cfDNA tumor fraction, in fact a significant difference in terms of cfDNA tumor fraction is observed between these two patients’ subgroup**


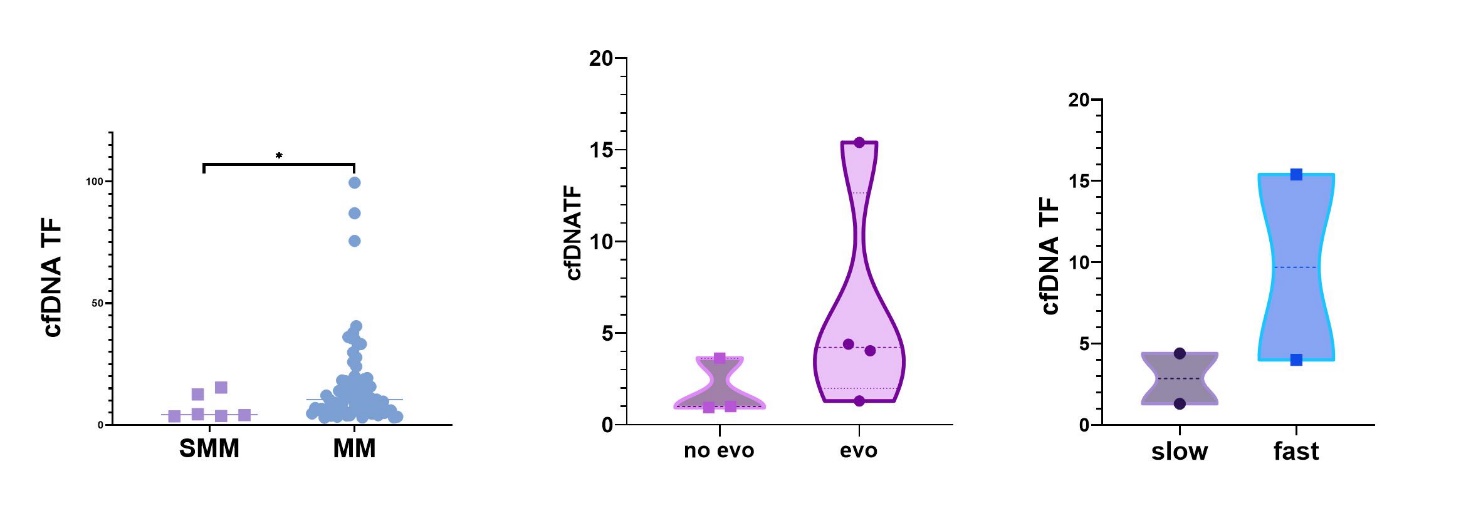


**Supplementary figure 2: cfDNA tumour fraction in preneoplastic phases**


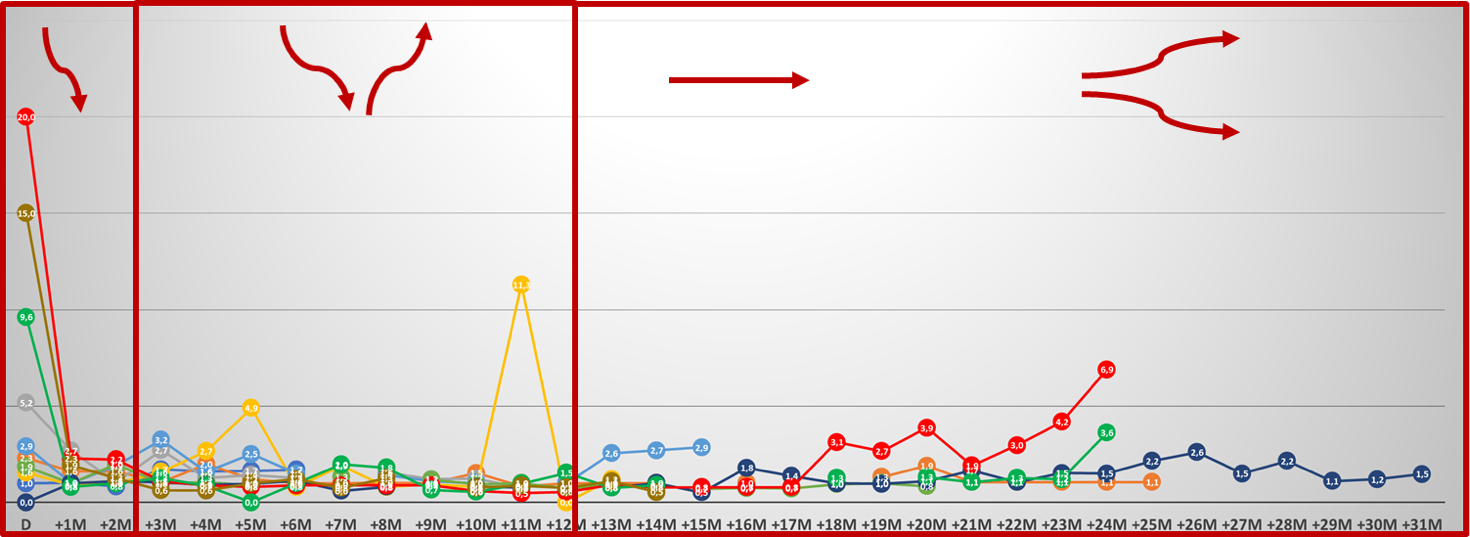


**Supplementary figure 3: cfDNA monitoring**

**
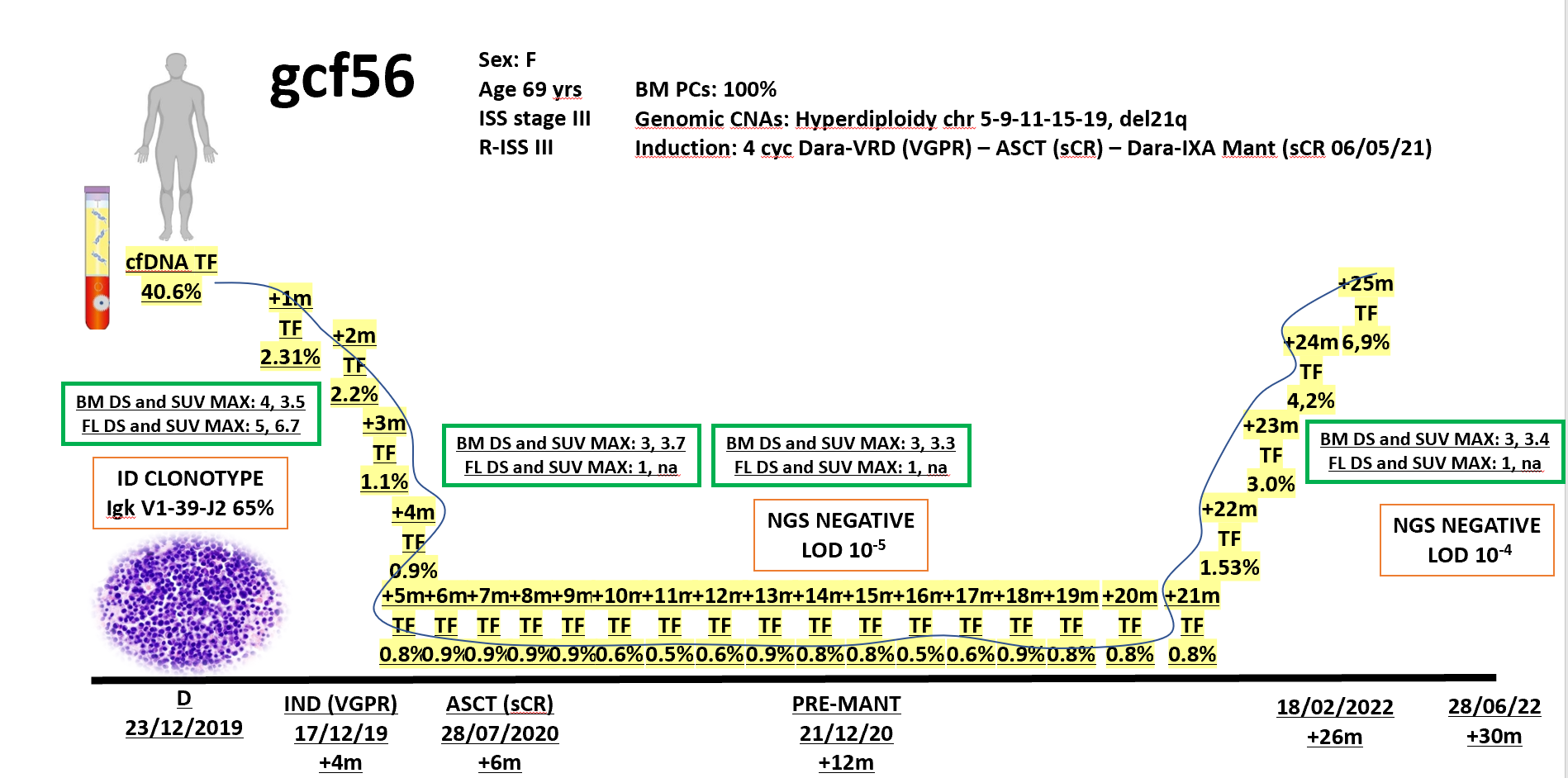
**

**Supplementary figure 4: Example of a patient monitored by a trimodality approach.** For example, patient *gcf56* showed a high cfDNA tumor load at baseline (TF = 40.6%), which dramatically decrease after induction therapy (up to 0.5%), and then, during maintenance treatment, cfDNA a few months ago it began to grow up to 7% of tumor fraction. This patient is now in complete remission, with both a negative PET scans and negative BM MRD and we will continue to monitor cfDNA tumor fraction in PB monthly, possibly anticipating any disease progression

**Supplementary Table 4. Example of a patient monitored by a trimodality approach.** Patient *gcf56* showed a high cfDNA tumor load at baseline (40.6%), which dramatically decreased under induction therapy (up to 0.5%), but that, during maintenance treatment, began to grow up to 7%. This patient, still in complete remission with both negative PET scans and negative BM MRD, is under active monitoring by monthly analysis of ctDNA, aiming at a possible anticipated detection of the disease progression.

| **MPC** | **gcf** | **MONTHS** | **TFgDNA_BM** | **TFctDNA_PB** | **CLINICAL FOLLOW-UP** |
| --- | --- | --- | --- | --- | --- |
| **MPC_269** | **gcf150** | **ID** | **31,85** | **15,70** | **DIAGNOSIS** |
|  |  | **7** |  | **8,1** | **FU1** |
|  |  | **12** |  | **46,87** | **FU2** |
|  |  | **24** | **99,61** | **91,9** | **RELAPSE** |

**Supplementary Table 5. cfDNA can anticipate a clinical progression.** Patient *gcf150*, whose gDNA and cfDNA were both profiled at baseline and who progressed after 24 months of therapy, a progressive ctDNA increase was detected at two subsequent time-points (from 8.1 at 7^th^ month, 46.8 at 12^th^ month to 91.9% at progression; p<.05), which might have predicted the clinical progression at least one year before its clinical assessment.

**Supplementary Table 6. Multivariate analysis of the impact of high ctDNA and cytogenetics on PFS.**

| **Variable** | **Class** | **high cfDNA TF** | **low cfDNA TF** | **n** | **p-value** | ***** |
| --- | --- | --- | --- | --- | --- | --- |
| **Calcio_M_105** | **0** | 19 | 63 | 82/98 | 0.036 | ***** |
|  | **1** | 8 | 8 | 16/98 | 0.036 | ***** |
| **HB_m_105** | **0** | 15 | 83 | 98/148 | 0.003 | ****** |
|  | **1** | 19 | 31 | 50/148 | 0.003 | ****** |
|  |  |  |  |  |  |  |
| **PC_M_60** | **0** | 10 | 66 | 76/117 | 0.015 | ***** |
|  | **1** | 14 | 27 | 41/117 | 0.015 | ***** |
| **R_ISS** | **1** | 7 | 32 | 39/87 | 0.006 | ****** |
|  | **2** | 13 | 25 | 38/87 | 0.006 | ****** |
|  | **3** | 7 | 3 | 10/87 | 0.006 | ****** |
|  |  |  |  |  |  |  |
| **Variable** | **Class** | **high cfDNA TF** | **low cfDNA TF** | **n** | **p-value** | ***** |
| **BM_DS≥4** | **0** | 16 | 54 | 70/96 | 0.05 |  |
|  | **1** | 11 | 15 | 26/69 | 0.05 |  |
| **EM** | **0** | 23 | 66 | 89/96 | 0.095 |  |
|  | **1** | 4 | 3 | 7/96 | 0.095 |  |
| **EM_DS** | **1** | 23 | 66 | 89/96 | 0.067 |  |
|  | **4** | 2 | 3 | 5/96 | 0.067 |  |
|  | **5** | 2 | 0 | 2/96 | 0.067 |  |
| **PS** | **0** | 13 | 56 | 70/96 | 0.005 | ******* |
|  | **1** | 14 | 13 | 26/96 | 0.005 | ******* |
| **PS_DS** | **1** | 14 | 56 | 70/96 | 0.003 | ******* |
|  | **3** | 2 | 3 | 5/96 | 0.003 | ******* |
|  | **4** | 4 | 8 | 12/96 | 0.003 | ******* |
|  | **5** | 7 | 2 | 9/96 | 0.003 | ******* |
| **n. FL** | **1** | 10 | 29 | 39/96 | 0.06 |  |
|  | **2** | 6 | 25 | 31/96 | 0.06 |  |
|  | **3** | 2 | 8 | 10/96 | 0.06 |  |
|  | **4** | 9 | 7 | 16/96 | 0.06 |  |
| **FL_DS** | **1** | 10 | 29 | 39/96 | 0.056 |  |
|  | **3** | 0 | 4 | 4/96 | 0.056 |  |
|  | **4** | 6 | 25 | 31/96 | 0.056 |  |
|  | **5** | 11 | 11 | 22/96 | 0.056 |  |
|  |  |  |  |  |  |  |
| **SUV MAX** |  |  |  | **corr** | **p-value** | ***** |
| **EM** |  |  |  | 0.269 | 0.008 | ****** |
| **PS** |  |  |  | 0.274 | 0.007 | ****** |
| **BM** |  |  |  | 0.185 | 0.073 |  |
| **FL** |  |  |  | 0.171 | 0.096 |  |

**Supplementary Table 7. Factors associated to high vs. low cfDNA TF/only imaging**

**Supplementary Table 8. Multivariate analysis of the impact of high ctDNA and PET/CT data on PFS.**

**
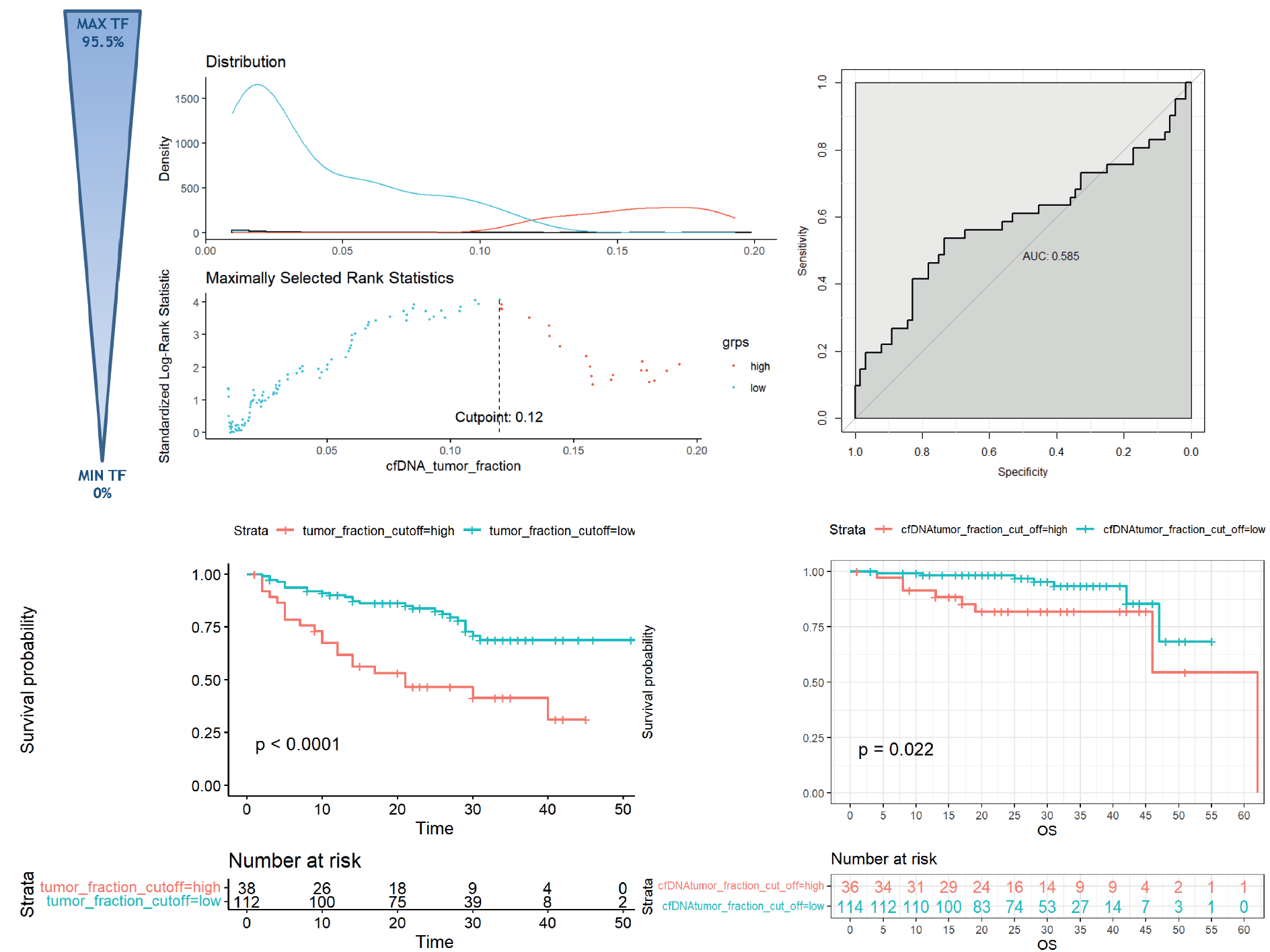
**

**Supplementary Figure 5. Identification of a cfDNA tumor fraction cut-off able to improve patients’ risk stratification**

**
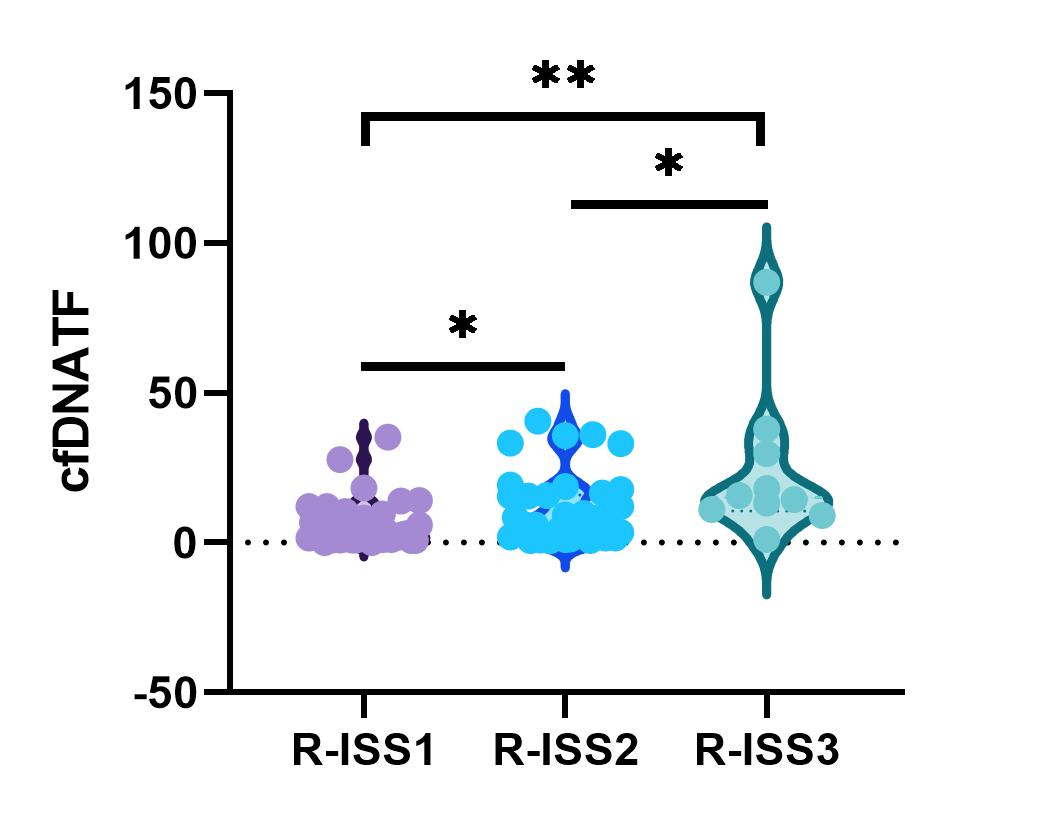
**

**Supplementary Figure 6. cfDNA TF according to R-ISS**


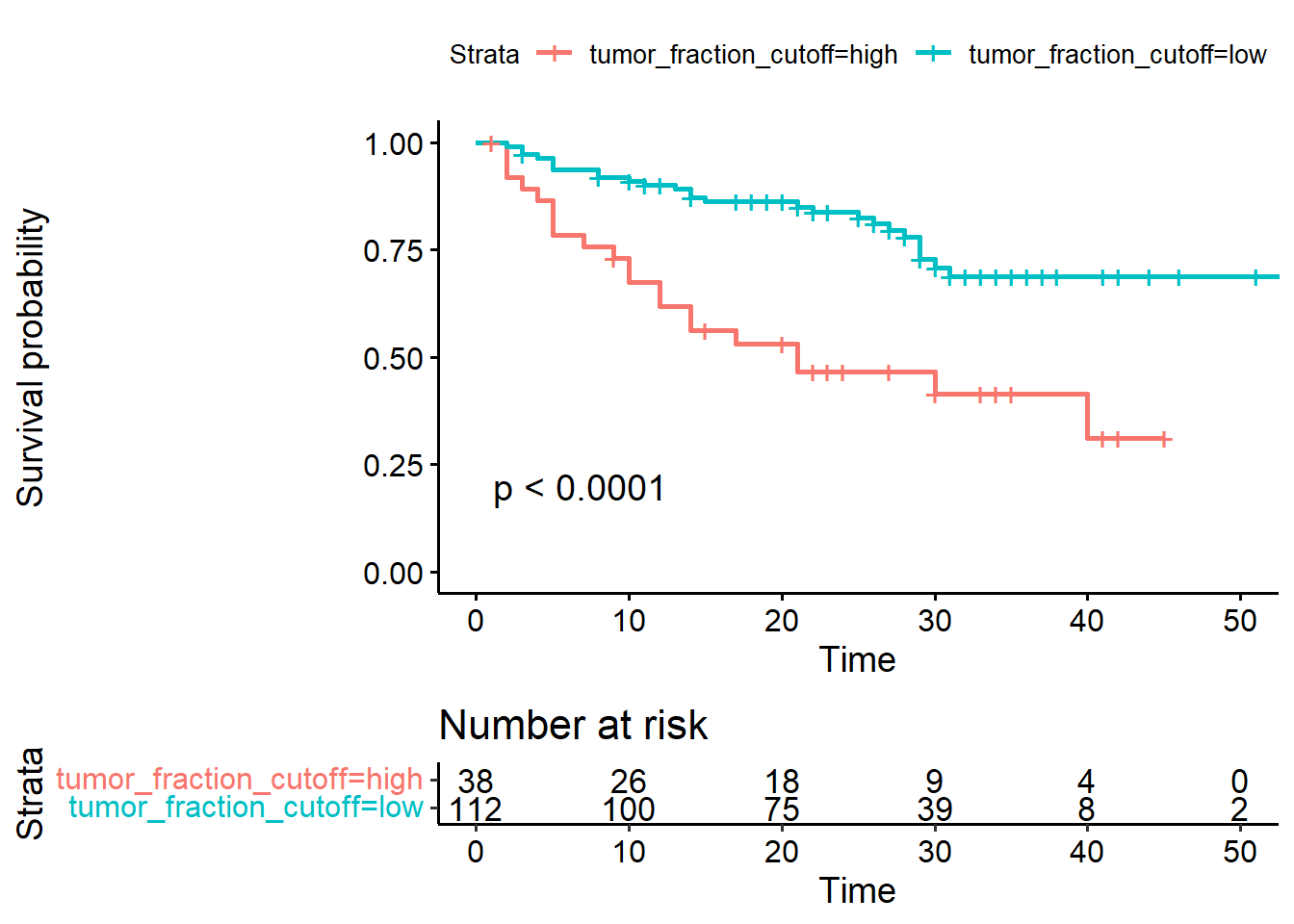

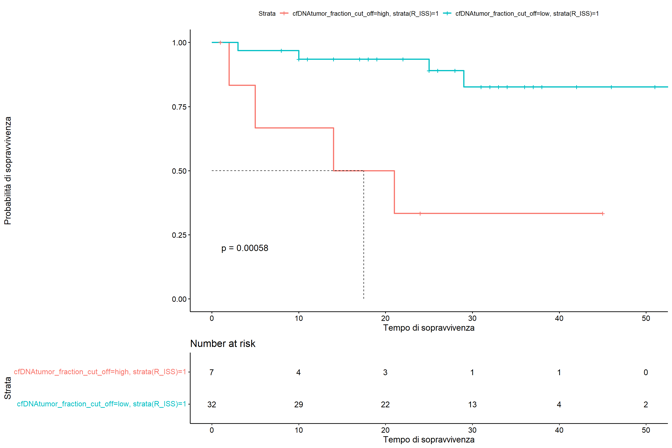


**Supplementary Figure 7. Kaplan Meyer curves representing the impact of cfDNA tumour fraction cut-off determination and PFS estimation of R-ISS I patients stratified according to the cfDNA TF cut-off.**

**
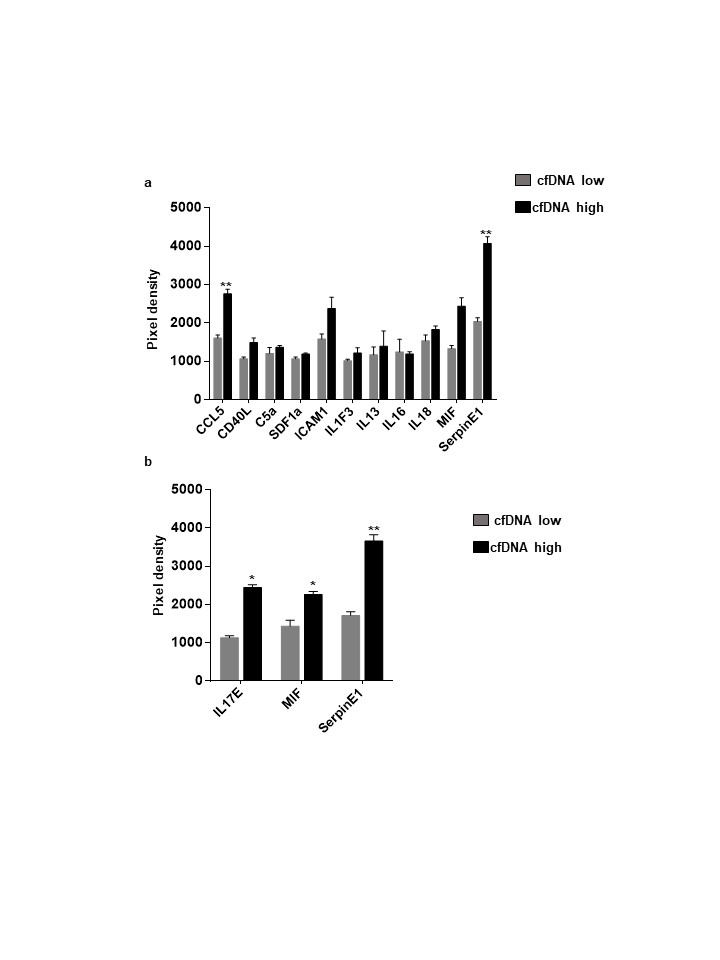
**

**Supplementary Figure 8. SerpinE1 expression is associated with poor prognosis in high cfDNA patients. (a)** Human Cytokine Array reveals differentially expression levels of CCL5 (p = 0.01), CD40L, C5a, SDF1α, ICAM1, IL1F3, IL13, IL16, IL18, MIF and SerpinE1 (p = 0.01) in bone marrow plasma from MM patients with cfDNA low (n= 8) and cfDNA high (n= 8). **(b)** IL17E (p = 0.003), MIF (p = 0.04) and SerpinE1 (p = 0.01) over-expression in fresh purified CAFs from the same patients reveals the role of SerpinE1 associated with tumor malignancy in cfDNA high patients. Array spots were analyzed with ImageJ Lab v. 1.51 software and normalized to positive control signal intensities. Graph bars represent the pixel density of the detected human cytokines in two independent experiments. Values are expressed as mean ± standard deviation of 16 independent experiments. Multiple unpaired t-test was performed.


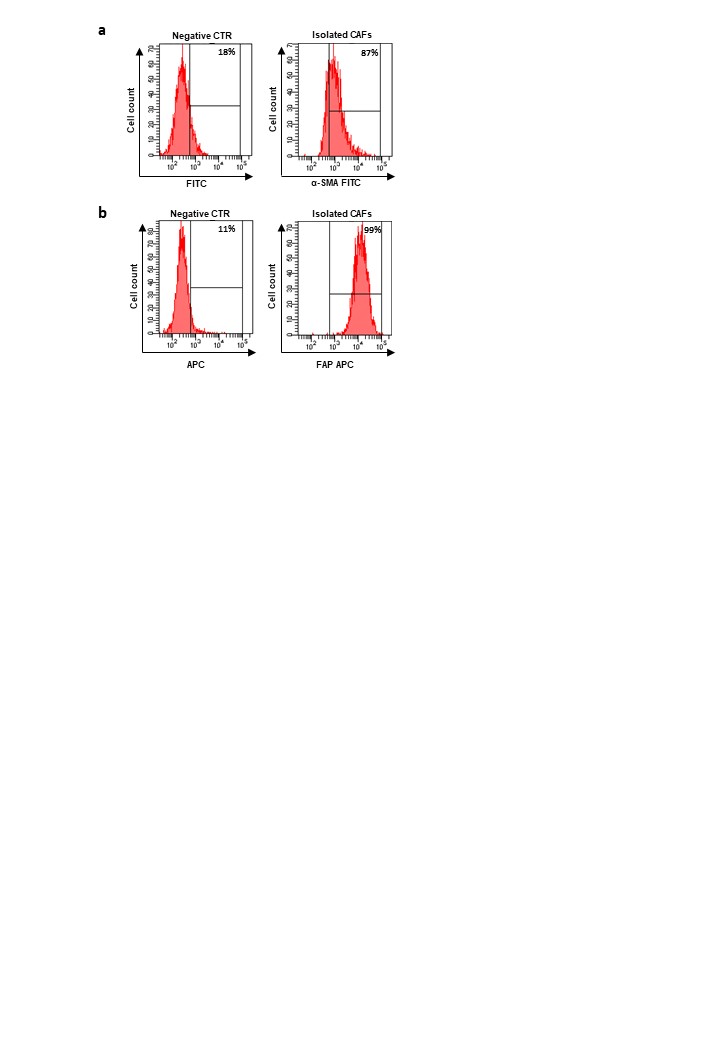


**Supplementary Figure 9**. **Cancer associated fibroblasts (CAFs) characterization.** (a-b) (CAFs) characterization by flow cytometry analysis of α-SMA and FAP expression in CAFs isolated from BMSCs of MM patients with high and low cfDNA. The cell purity (93%) of CAFs population was determined using the FACScanto II cytofluorimetry system (Becton Dickinson-BD, San Jose, CA, USA).

**
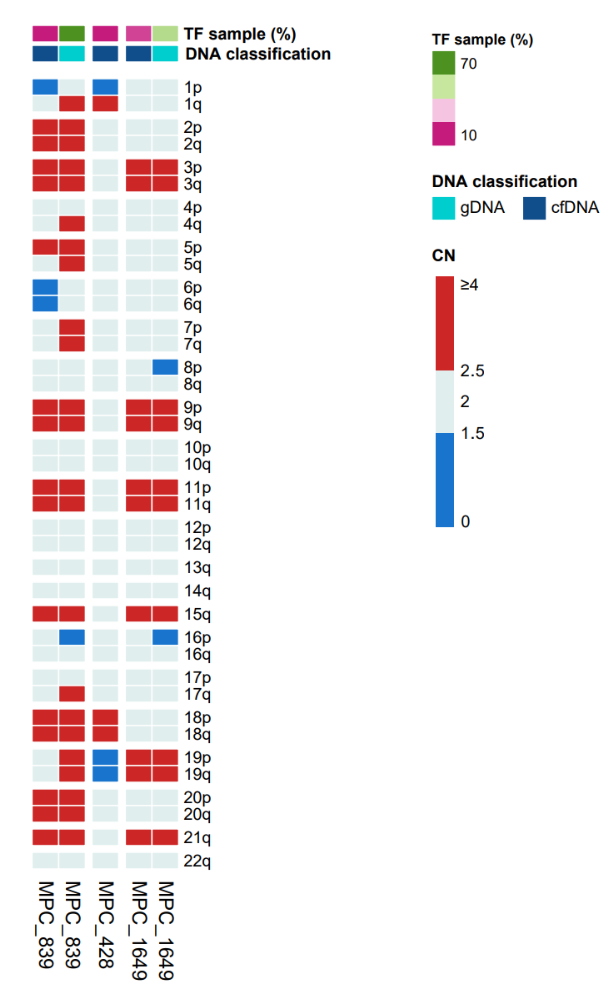
**

**Supplementary figure 10. cfDNA utility in SPC cases, where the bone marrow aspirates from iliac crest is not informative and its cellularity is usually modest.** Chromosome copy number alterations overview of three exemplary cases of SPC, where cfDNA permits the identification of the whole genome CNVs. 4 out of 7 cases (57.1%) resulted evaluable for CNVs detection in cfDNA, although the rest of the patients remains evaluable only on gDNA.

28. Martello, M., Remondini, D., Borsi, E., Santacroce, B., Procacci, M., Pezzi, A., . . . et al. (2016). Opposite activation of the Hedgehog pathway in CD138+ plasma cells and CD138-CD19+ B cells identifies two subgroups of patients with multiple myeloma and different prognosis. Leukemia, 30(9), 1869-1876. doi:10.1038/leu.2016.77

29.Boellaard R, Delgado-Bolton R, Oyen WJ, et al: FDG PET/CT: EANM procedure guidelines for tumor imaging: Version 2.0. Eur J Nucl Med Mol Imaging 42:328-354, 2015

30.Nanni C, Versari A, Chauvie S, et al: Interpretation criteria for FDG PET/CT in multiple myeloma (IMPeTUs): Final results—IMPeTUs (Italian Myeloma Criteria for PET USe). Eur J Nucl Med Mol Imaging 45:712-719, 2018

31.Bezzi D, Ambrosini V, Nanni C. Clinical Value of FDG-PET/CT in Multiple Myeloma: An Update. Semin Nucl Med. 2023 May;53(3):352-370. doi: 10.1053/j.semnuclmed.2022.10.008. Epub 2022 Nov 26. PMID: 36446644.

32.Frassanito MA, De Veirman K, Desantis V, Di Marzo L, Vergara D, Ruggieri S, … et al. Halting pro-survival autophagy by TGFβ inhibition in bone marrow fibroblasts overcomes bortezomib resistance in multiple myeloma patients. Leukemia. 2016 Mar;30(3):640-8. doi: 10.1038/leu.2015.289. Epub 2015 Oct 21. PMID: 26487273.
